# A dual A-to-Y and G-to-Y base editor and a four-base concurrent hypermutator in mammalian cells

**DOI:** 10.1038/s41467-026-77241-5

**Published:** 2026-08-27

**Authors:** Na Zhao, Joshua Seun Olajide, Zhihong Guan, Junyun Huang, Xiubin Zou, Feilong Ou, Jiejing Lin, Tianfu Tao, Tinghai Zhou, Zongliang Gao, Zemin Ren, Xiaoxia Shi, Xiaotian Huang, Gang Wang

**Affiliations:** 1 Key Laboratory of Prevention and Treatment of Cardiovascular and Cerebrovascular Diseases, Ministry of Education; School of Basic Medical Sciences, Gannan Medical University, Ganzhou, China; 2 Ganzhou Key Laboratory of Microbiology and Precision Diagnosis and Treatment; Department of Laboratory Medicine, First Affiliated Hospital of Gannan Medical University, Ganzhou, China; 3 Guangzhou JinHua JiYin (进化基因) Technology Co., Ltd., Guangzhou, China; 4 Precise Genome Engineering Center, School of Life Sciences, Guangzhou University, Guangzhou, China; 5 GMU-GIBH Joint School of Life Sciences, The Guangdong-Hong Kong-Macau Joint Laboratory for Cell Fate Regulation and Diseases, Guangzhou Medical University, Guangzhou, China; 6 Department of Basic Medical Sciences, Wuxi School of Medicine; Jiangnan University, Wuxi, China; 7 School of Life Sciences, Liaoning Provincial Key Laboratory of Biotechnology and Drug Discovery, Liaoning Normal University, Dalian, China; 8 The Second Affiliated Hospital of Nanchang University, School of Basic Medical Sciences, Jiangxi Medical College, Nanchang University, Nanchang, China

**Keywords:** CRISPR-Cas9 genome editing, CRISPR-Cas9 genome editing

## Abstract

Current dual base editors and hypermutators exhibit limited types of base conversion and constrained mutational diversity. To address this challenge, we engineer a compact dual base editor “A&GBE” by fusing a deaminase and glycosylase with nickase Cas9 (nCas9) to enable concurrent adenine and cytosine editing. Furthermore, we develop quadruple base mutating modules (QBMM) by fusing nickase or dead Cas9 with engineered thymine-DNA glycosylase (TDG), N-methylpurine DNA glycosylase (MPG) and TadA8e, together with MS2-MCP system recruited activation-induced cytidine deaminase (AID). This QBMM enables simultaneous mutation of all four types of nucleotide bases (A, T, G, C) within an approximately 100 base pair (bp) sequence surrounding the gRNA target site. This platform generates hyper diverse multi-nucleotide variants, establishing a powerful tool for accelerated protein evolution, functional genomics, and disease modeling.

## Abstract (web summary)

Current base editors exhibit limited types of base conversion and constrained mutational diversity. Here the authors develop a dual base editor for A-to-Y and G-to-Y changes, and a quadruple base mutating module (QBMM) that mutates all four DNA bases in mammalian cells.

## Introduction

Several single-nucleotide base editors (BEs) have been developed, including cytosine base editors (CBEs) for C-to-T conversion^1^, adenine base editors (ABEs) for A-to-G transition^2^, C-to-G base editors (CGBEs)^3,4^, adenine-to-pyrimidine base editors (AYBEs)^5^, guanine-to-pyrimidine base editors (GYBEs)^6^, and T-to-G base editors (TGBEs)^7–9^. To broaden their editing capability, several dual base editors have been developed to enable a simultaneous editing of two distinct base types, such as A-to-G and C-to-T^10–13^, A-to-G and C-to-G^14,15^, or A-to-G/C/T and C-to-T/G/A^16^. These dual base editors have great potential for creating or reverting multi-nucleotide variants (MNVs)^17,18^, an emerging category of sequence variants associated with genetic diseases. While current AYBE and GYBE facilitate targeted pyrimidine transversions, they have a restricted editing scope that limits their applications and therefore require multiplexed editing for the correction of diverse mutations. To address this, we engineer a dual-functional adenine and guanine base editor (A&GBE), which achieves simultaneous A-to-Y and G-to-Y conversions within a single editing window. However, even the most advanced dual base editors, including A&GBE, are inherently limited to modifying only two nucleotide types, thereby leaving the majority of possible base substitutions inaccessible.

Beyond generating or reverting MNVs, dual base editors and hypermutators represent powerful tools for directed evolution in mammalian systems. However, there are no optimal mutators available for directed evolution in mammalian cells that satisfy two fundamental parameters—high mutation rates and an unbiased mutational spectrum. Two CRISPR-activation-induced cytosine deaminase (CRISPR-AID)-based hypermutators have been demonstrated with high mutation rates for genomic diversification in mammalian cells^19,20^. However, their utility remains restricted due to pronounced C/G bias—predominantly converting cytosine and guanine bases while largely preserving adenine and thymine nucleotides^19,20^. Although emerging dual base editors partially address the limitation through expanded editing profiles, the current systems still fail to achieve comprehensive diversification across all four DNA bases. Thus, the field urgently needs a single tool capable of simultaneously targeting all nucleotides to achieve unbiased, high-efficiency mutagenesis.

To overcome these challenges, we develop a quadruple-base-mutating module (QBMM), which uniquely integrates an adenine deaminase (TadA8e), a thymine-DNA glycosylase (TDG), a cytosine deaminase (AID), and an engineered guanine excision protein (MPG) to concurrently mutate adenine, thymine, cytosine, and guanine within a single editing window. In marked contrast to existing base editors that are confined to specific base transitions or transversions, and unlike hypermutators that exhibit strong nucleotide bias, QBMM achieves a comprehensive and well-balanced mutational spectrum across all four DNA bases. This design directly addresses the two critical bottlenecks in CRISPR-based base editing—limited editing scope and skewed mutational profiles—and fulfills the long-standing need for a mutator that simultaneously delivers high mutation rates and unbiased diversification. QBMM therefore serves as a transformative platform for directed evolution and protein engineering in mammalian cells.

## Results

### Development of a dual base editor (A&GBE) for adenine and guanine editing

**Fig. 1:**
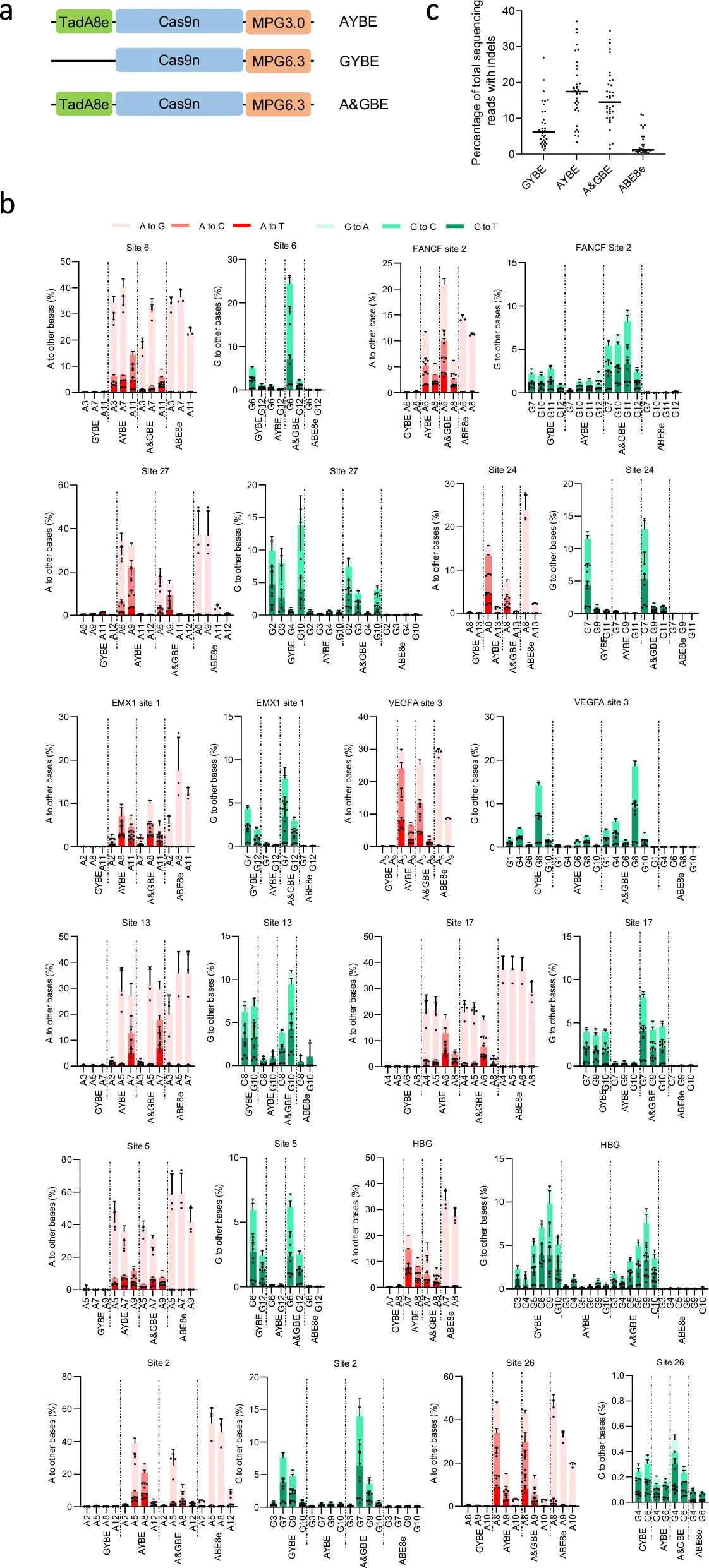
Development of a dual base editor for simultaneous adenine and guanine editing. **a** Schematic architectures of AYBE, GYBE, and A&GBE. **b** Editing activities of AYBE, GYBE, and A&GBE at 12 endogenous genomic sites. **c** Cumulative indel frequencies across all sites (see Supplementary Fig. 4 for detailed indel information). Values and error bars reflect the mean and s.d. of three biological replicates performed on separate days. Source data are provided as a Source Data file.

AYBE^5^ enables A-to-Y(C/T) editing via a fusion protein comprising an adenine deaminase (TadA8e), nCas9, and an engineered hypoxanthine excision protein MPG3.0 (MPG carrying G163R, N169S, S198A, K202A, G203A, S206A and K210A mutations). Additionally, GYBE^6^ mediates G-to-Y editing using nCas9 fused to a further engineered version, MPG6.3 (MPG3.0 carrying S169G, D175R, C178N, and Q294R mutations), which excises guanine. Given the structural similarity between deoxyinosine (the deamination product of adenine) and guanine, we hypothesized that MPG6.3 could catalyze the excision of both deoxyinosine and guanine. This dual-substrate capability would enable a single editor to simultaneously target both A and G bases, thereby expanding the scope of base editing for concurrent A and G conversions. To test this, we engineered a dual base editor, A&GBE, by fusing TadA8e, nCas9, and MPG6.3 (Fig. 1a). Comparative analysis at 12 endogenous loci in HEK293T cells revealed that A&GBE maintained robust dual-activity, achieving both A-to-Y and G-to-Y editing at all tested sites (Fig. 1b). In contrast, control editors (AYBE and GYBE) exhibited strictly mono-specific activities, consistent with prior reports^5,6^. Further comparison revealed that AYBE exhibited higher A-to-Y editing activity than A&GBE at most genomic sites (except ABE site 13, FANCF site 2), whereas A&GBE displayed higher G-to-Y editing activity than GYBE at most genomic sites (except ABE site 27, HBG), implying enhanced guanine excision when coupled with TadA8e. Notably, at TAG motifs, A&GBE outperformed AYBE in A-to-Y editing, while at TGA and CGA motifs, it exhibited even more pronounced G-to-Y editing relative to GYBE (Supplementary Fig. 1). Compared with AYBE, A&GBE showed lower A-to-T and A-to-C editing activity and a narrower editing window, but higher A-to-G editing. Compared with GYBE, A&GBE displayed higher overall G editing, particularly G-to-A and G-to-C (Supplementary Figs. 2 and 3). The elevated indel frequencies observed with AYBE and GYBE are consistent with the established mechanism^6^, whereby MPG-mediated base excision and subsequent AP endonuclease cleavage, combined with the nCas9-induced nick, produce DNA double-strand breaks. Consistent with this mechanism, A&GBE also induced substantial indels, likely reflecting the robust deoxyinosine (from A deamination) and guanine excision activity of MPG6.3. Notably, the overall indel frequencies of A&GBE were slightly lower than those of AYBE (Supplementary Fig. 4).

### Narrowing the editing window of AYBE and A&GBE

**Fig. 2:**
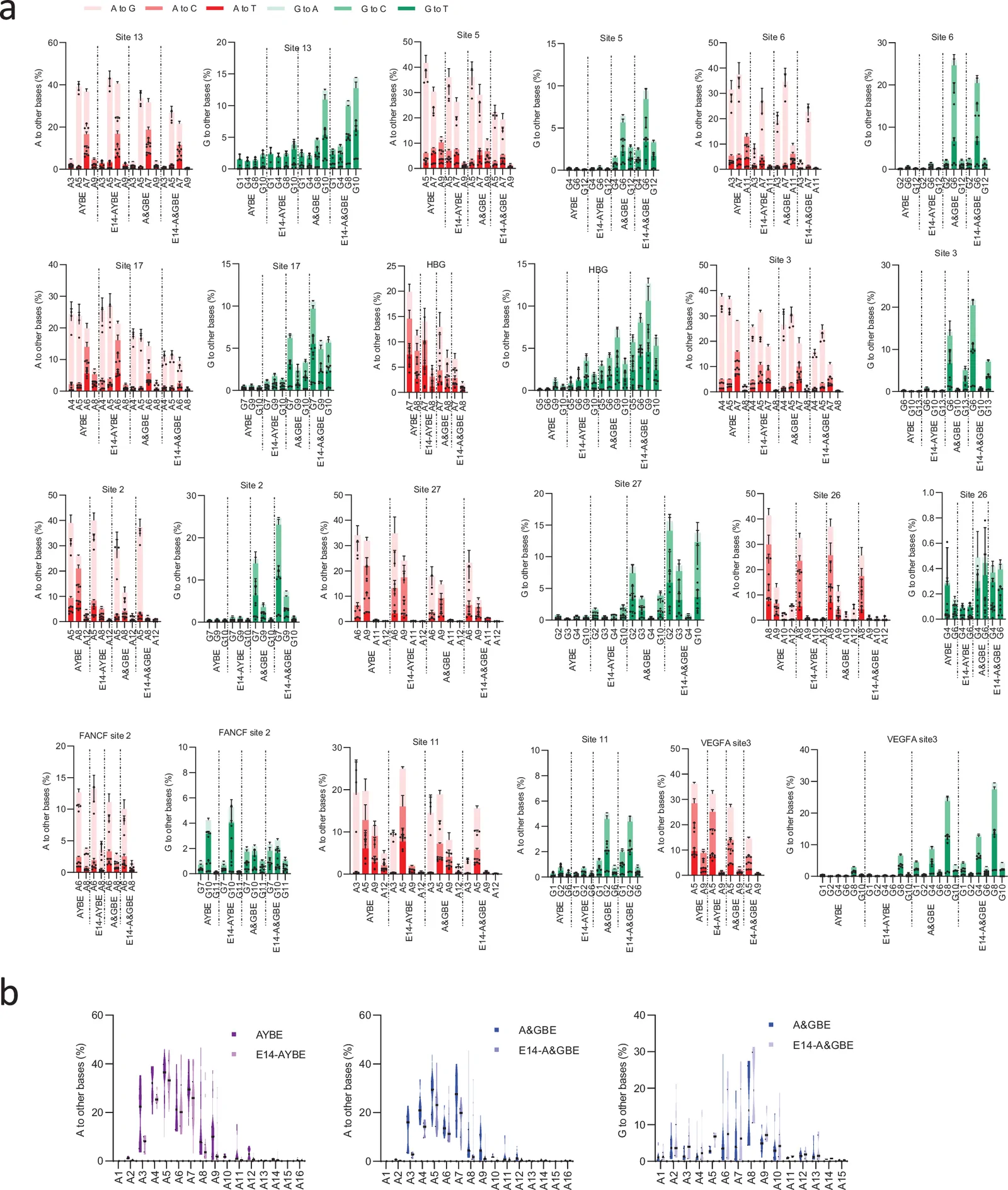
AYBE and A&GBE, with previously evolved adenine deaminase, exhibit narrowed editing windows. **a** Editing profiles of AYBE, E14-AYBE, A&GBE, and E14-A&GBE at 12 genomic sites. Values and error bars represent the mean and s.d. from three biological replicates performed on separate days (see Supplementary Fig. 6 for indel information). **b** Cumulative frequencies of A to other bases and G to other bases across the protospacer at the edited sites (PAM located at positions 21–23). Source data are provided as a Source Data file.

Narrowing the editing window enables exclusive targeting of a single base, thereby ensuring precise modification of the intended base. To constrain the broad editing window of AYBE and A&GBE (both employing the TadA8e^21^ adenine deaminase, which has a broad editing window), we generated six variants by replacing the TadA8e domain with our previously characterized adenine deaminase variants—E4-TadA, E14-TadA, and E11-TadA^22^, yielding E4-AYBE, E14-AYBE, and E11-AYBE for AYBE, and E4-A&GBE, E14-A&GBE, and E11-A&GBE for A&GBE (Supplementary Fig. 5a). We first evaluated the editing profiles of all six variants alongside the original AYBE, A&GBE, and ABE8e at six genomic loci (Site 13, Site 17, HBG, Site 5, Site 6, Site 26) (Fig. 2a and Supplementary Fig. 5b). All AYBE-based variants (E4-, E14-, and E11-AYBE) exhibited a narrowed adenine-editing window compared with parental AYBE (Fig. 2b), consistent with our previous observations^22^. Among these variants, E14-AYBE maintained editing efficiency comparable to AYBE, whereas E4-AYBE and E11-AYBE showed reduced activity. Similarly, all three A&GBE variants (E4-, E14-, and E11-A&GBE) exhibited a narrowed adenine-editing window relative to parental A&GBE. Among them, E14-A&GBE largely maintained editing activity, whereas E4-A&GBE and E11-A&GBE showed compromised efficiency. To further verify these editing properties across a broader set of targets, we tested the two best-performing variants, E14-AYBE and E14-A&GBE, together with the original AYBE and A&GBE, at an additional six loci (Site 3, Site 2, Site 27, FANCF site 2, Site 11, and VEGFA site 3) (Fig. 2a and Supplementary Figs. 5c). At these loci, both E14-AYBE and E14-A&GBE maintained narrowed adenine-editing windows. Notably, across 8 out of the total 12 genomic sites examined (Site 13, Site 17, HBG, Site 5, Site 3, Site 2, Site 27, and VEGFA site 3), the E14-A&GBE showed higher guanine editing activity and a broader editing window than the parental A&GBE (Fig. 2a, b).

### Comparison of editing activity between nCas9 and dCas9-derived AYBE, GYBE, and A&GBE

**Fig. 3:**
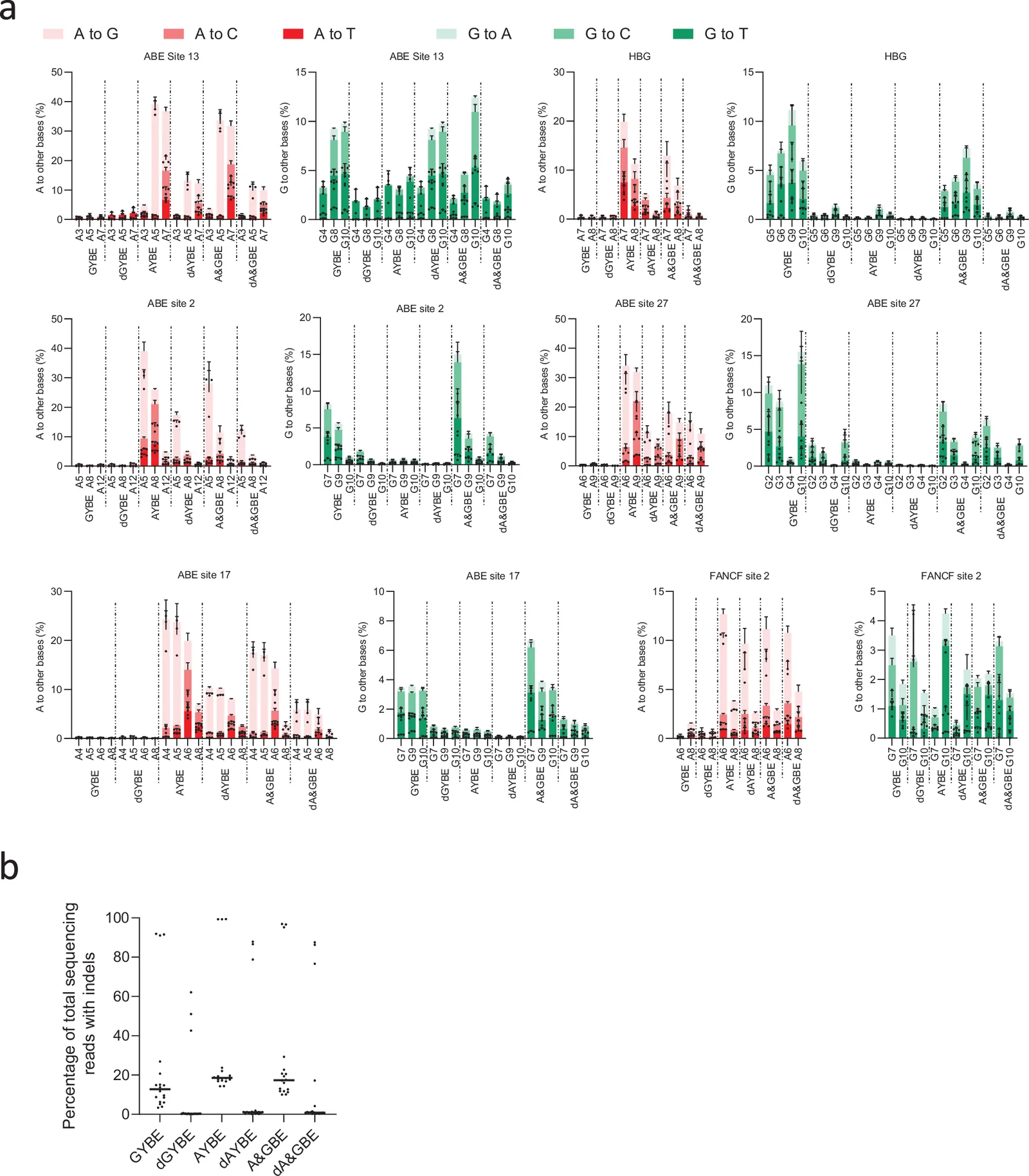
Comparison of AYBE, GYBE, and A&GBE editing based on nCas9 and dCas9. **a** Editing activities of AYBE and A&GBE at six genomic sites. **b** Cumulative indel frequencies across all sites (see Supplementary Fig. 9 for detailed indel information). Values and error bars reflect the mean and s.d. of three biological replicates performed on separate days. Source data are provided as a Source Data file.

The previously characterized AYBE^5^ and GYBE^6^ were based on nCas9, which is largely due to the fact that the nicking property of nCas9 mediated higher base editing compared to that of dCas9, as initially demonstrated for CBE and ABE. However, nCas9 also induced a higher indel rate, an aspect that poses a challenge to therapeutic applications. The performance of dCas9-derived AYBE and GYBE remained unexplored. To address this, we investigated whether replacing dCas9 with nCas9 in AYBE, GYBE, and our dual-base editor A&GBE would similarly reduce indels. We constructed three dCas9-derived editor variants (dGYBE, dAYBE, and dA&GBE) and compared their editing profiles with nCas9-derived variants, with ABE8e included as a control, in HEK293T cells. As with prior studies^5,6^ and our earlier findings, AYBE, GYBE, and A&GBE showed adenine, guanine, and concurrent adenine and guanine editing activity respectively, at six genomic sites (Fig. 3 and Supplementary Figs. 7–9). dGYBE exhibited significantly reduced guanine-editing efficiency relative to GYBE and showed markedly lower indel frequencies. dAYBE demonstrated lower adenine-editing activity than AYBE, yet achieved a pronounced reduction in indel rates. dA&GBE displayed decreased dual-base-editing efficiency, but substantially reduced indels compared with A&GBE. Overall, all three dCas9-derived editors exhibited significantly lower indel frequencies than their nCas9 counterparts. In addition, we observed diverse editing profiles for A&GBE and dA&GBE (Supplementary Fig. 10), which could be leveraged to develop a hypermutator.

### Development of a triple-base-mutating module by the combinatorial use of A&GBE and AID

**Fig. 4:**
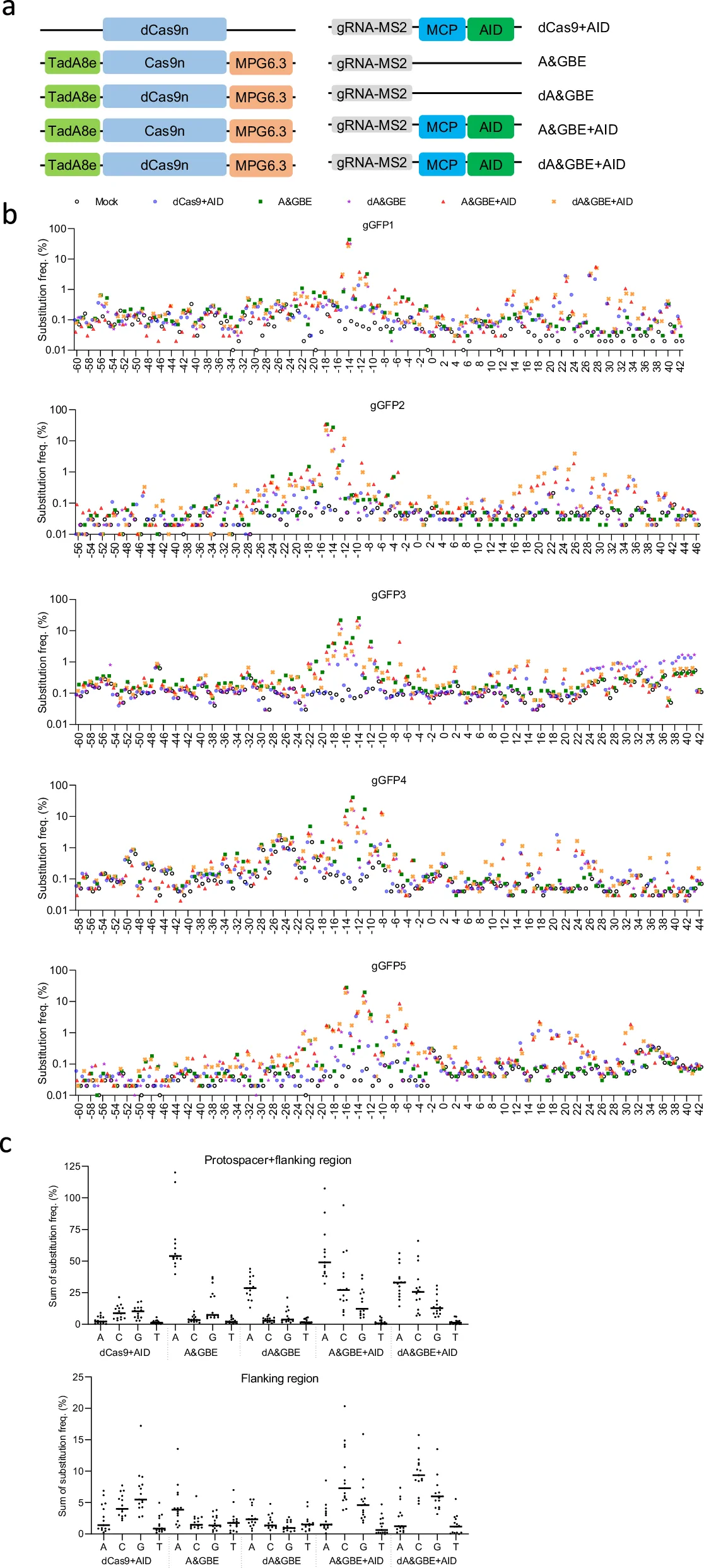
Development of mutators inducing mutations on adenine, guanine, and cytosine. **a** Schematic of mutator design. **b** Distribution of base substitution frequencies along the GFP sequence. Frequency (freq.) at each base was calculated as the percentage of reads containing substitutions relative to the total reads covering that base (%). Each dot represents a nucleotide. Data from the first of three independent biological replicates are shown (see Supplementary Fig. 12 for all three repeats). **c** The total base substitution frequency (Mock-subtracted at each nucleotide position) within the protospacer (−20 to 0) and flanking regions, as well as for the flanking region alone. Each data point represents one of five independent GFP target sites from one of three biological replicates (*n* = 15 independent transfections). Horizontal bars indicate the median. Source data are provided as a Source Data file.

Based on A&GBE and dA&GBE, we sought to develop an advanced base mutator capable of simultaneously editing adenine, guanine, and cytosine. Given that AID (hyperactive cytosine deaminase) is known to primarily mutate cytosine and guanine^19,20^, we hypothesized that combining A&GBE or dA&GBE with AID could achieve this goal. Unlike A&GBE or dA&GBE, AID was recruited to the target site via the MS2-MCP system (Fig. 4a). We evaluated the mutation activity of dA&GBE + AID and A&GBE + AID in a GFP-integrated HEK293T cell line (Fig. 4a, see Supplementary Fig. 11 for workflow). dCas9+AID, A&GBE, and dA&GBE were also included for testing (Fig. 4b and Supplementary Fig. 12). With minimal substitution frequencies (mostly <0.1%) for the blank control, dCas9+AID displayed 1-5% substitution frequencies across all five tested GFP target sites (gGFP1-5), consistent with a previous report^20^. Within the protospacer regions (position −20 to 0, where position 0 is adjacent to the PAM) of the five gRNAs, we observed varying nucleotide substitution frequencies for A&GBE editors. Both dA&GBE + AID and A&GBE + AID induced comparable levels of mutagenesis throughout the target region, exceeding the activity of dA&GBE, A&GBE, and dCas9+AID at the majority of positions. Interestingly, constructs with AID showed higher substitution frequencies in a 40-bp PAM-adjacent flanking region (position 0 to 40) across all five gRNAs targeting sites.

Next, we analyzed the mutational signatures of each construct by quantifying base conversion frequencies across the protospacer and flanking regions (Fig. 4c). Consistent with prior reports^19^, dCas9+AID preferentially converted C or G to other bases. In contrast, A&GBE and dA&GBE primarily induced A or G conversions, aligning with our earlier findings. Strikingly, A&GBE + AID and dA&GBE + AID generated high-frequency substitutions at A, C, and G, demonstrating triple-base-editing capability. To assess mutational patterns beyond the protospacer, we calculated the median frequencies of base conversions in the flanking regions (Fig. 4c). Here, dCas9+AID maintained elevated C/G substitutions, whereas A&GBE and dA&GBE showed stronger A-specific activity without significant G editing. Notably, A&GBE + AID and dA&GBE + AID outperformed all other constructs in C/G substitution rates but exhibited unexpectedly low A editing in these regions. Both A&GBE + AID and dA&GBE + AID exhibited more evenly distributed C and G mutations to other bases compared with A&GBE and dA&GBE (Supplementary Fig. 13). Given the advantages of dA&GBE + AID for downstream applications, we selected it for further development of a quadruple-base mutation system.

### Development of a quadruple base-mutating module (QBMM) for four-base editing

**Fig. 5:**
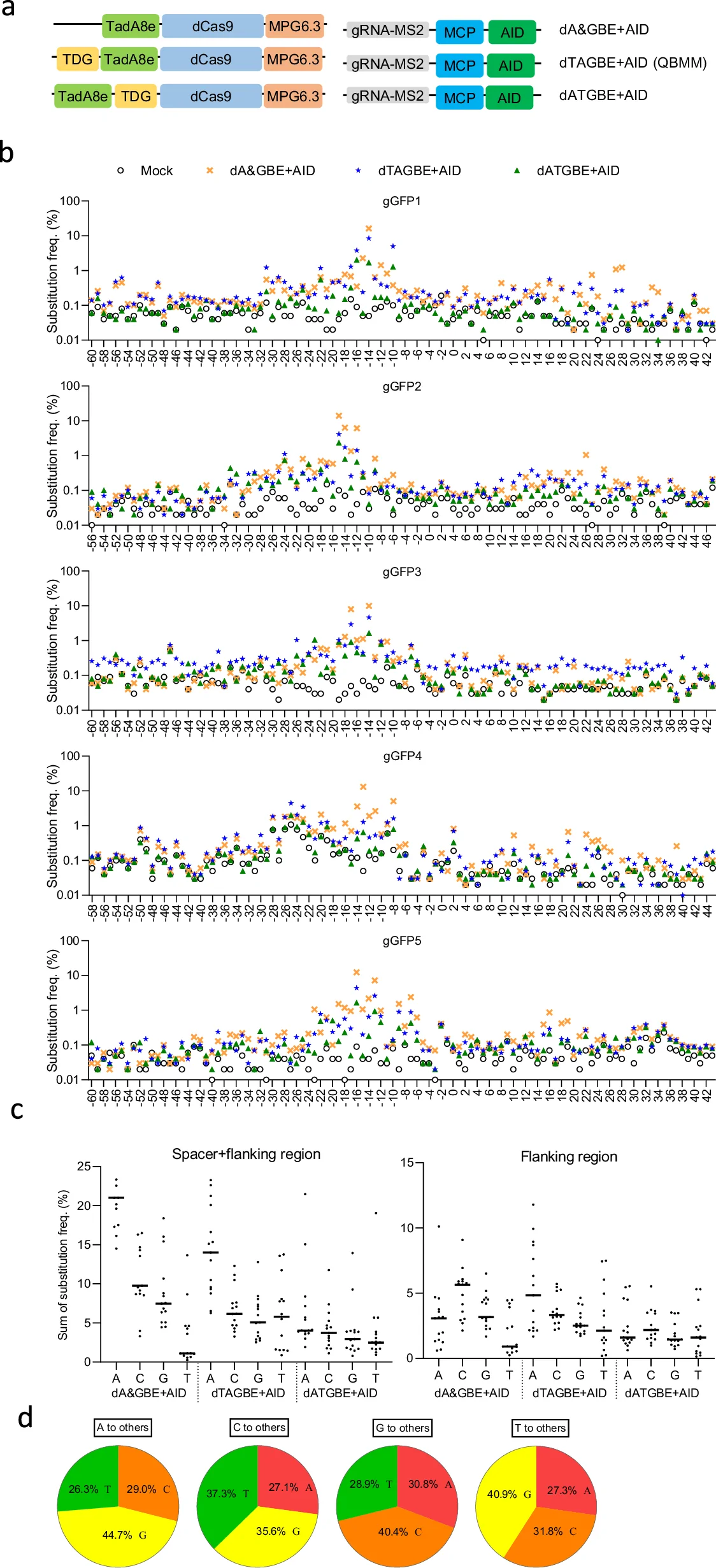
Development of mutators for all four bases. **a** Schematic design of mutators. **b** Distribution of base substitution frequencies along the GFP sequence. Frequency at each position was calculated as the percentage of reads containing a substitution relative to all reads covering that base. Each dot represents a nucleotide. Data from the first repeat of three independent biological replicates are shown (see Supplementary Fig. 14 for all three repeats). **c** Total base substitution frequency (Mock-subtracted at each nucleotide position) calculated for the protospacer region (positions −20 to 0) plus flanking regions, and for the flanking regions alone. Each data point represents one of five independent GFP target sites from one of three biological replicates (*n* = 15 independent transfections). Horizontal bars indicate the median. **d** Proportions of A, C, G, and T mutated to other bases by dTAGBE+AID (see Supplementary Fig. 15 for other mutators). Data are from three independent experiments. Source data are provided as a Source Data file.

Although thymine-DNA glycosylase (TDG) has been engineered as a T-to-G base editor (TGBE)^7–9^, its activity also induces T-to-A and T-to-C conversions through thymine excision followed by base excision repair (BER). We hypothesized that integrating engineered TDG^8^ into the dA&GBE + AID module could further expand both mutagenic scope and efficiency. Based on TadA8e-dCas9-MPG, we generated two mutator constructs (TDG-TadA8e-dCas9-MPG + AID and TadA8e-TDG-dCas9-MPG + AID, abbreviated as dTAGBE+AID and dATGBE+AID respectively). We assessed the editing efficiency of the three mutator groups in GFP-integrated HEK293T cells (Fig. 5a, see Supplementary Fig. 9 for workflow). Deep sequencing results showed that A&GBE + AID and dTAGBE+AID exhibited comparable substitution frequencies across all five targeted protospacer and flanking regions, and both were generally higher than those of dATGBE+AID (Fig. 5b and Supplementary Fig. 14).

We then analyzed the mutational profiles of these mutator groups in detail for the protospacer and flanking regions (Fig. 5c). Compared with dA&GBE + AID, dTAGBE+AID and dATGBE+AID introduced higher levels of T substitution in both the combined protospacer and flanking regions and in the flanking regions alone, confirming functional TDG integration. dTAGBE+AID demonstrated superior editing efficiency compared with dATGBE+AID in both region sets, indicating that the relative positioning of the deaminase and glycosylase significantly influenced editing outcomes. Although dTAGBE+AID generated less A, C, and G editing than dA&GBE + AID in the combined protospacer and flanking regions, it unexpectedly generated higher A and T substitutions in the flanking regions. Overall, dTAGBE+AID produced a more balanced mutational spectrum across all four bases, with particularly A-to-other base conversions compared with dA&GBE + AID (Fig. 5d and Supplementary Fig. 15). Collectively, dTAGBE+AID achieved high substitution frequencies with an expanded activity window, enabling comprehensive mutagenesis of all four bases. dTAGBE+AID is therefore recommended for future applications.

### Comparison of cell viability among base editors

To evaluate whether stacking multiple modules (AID, TadA8e, TDG, MPG) exacerbates BER-related genotoxicity, we compared cell viability among AID, AGBE, dA&GBE, A&GBE + AID, dA&GBE + AID, and dTAGBE using the CCK8 assay at 72 h post-transfection, with the mock group set to 100% (Supplementary Fig. 16). AID alone exhibited negligible cytotoxicity. While all A&GBE-containing constructs led to reduced cell viability, dA&GBE and dA&GBE + AID showed improved viability compared with their A&GBE and A&GBE + AID counterparts respectively, indicating that dCas9-based configurations confer lower cytotoxicity than those utilizing nCas9 alone. dTAGBE +AID displayed the lowest viability among the tested groups.

### QBMM enables the identification of MEK1 inhibitor-resistance mutations

**Fig. 6:**
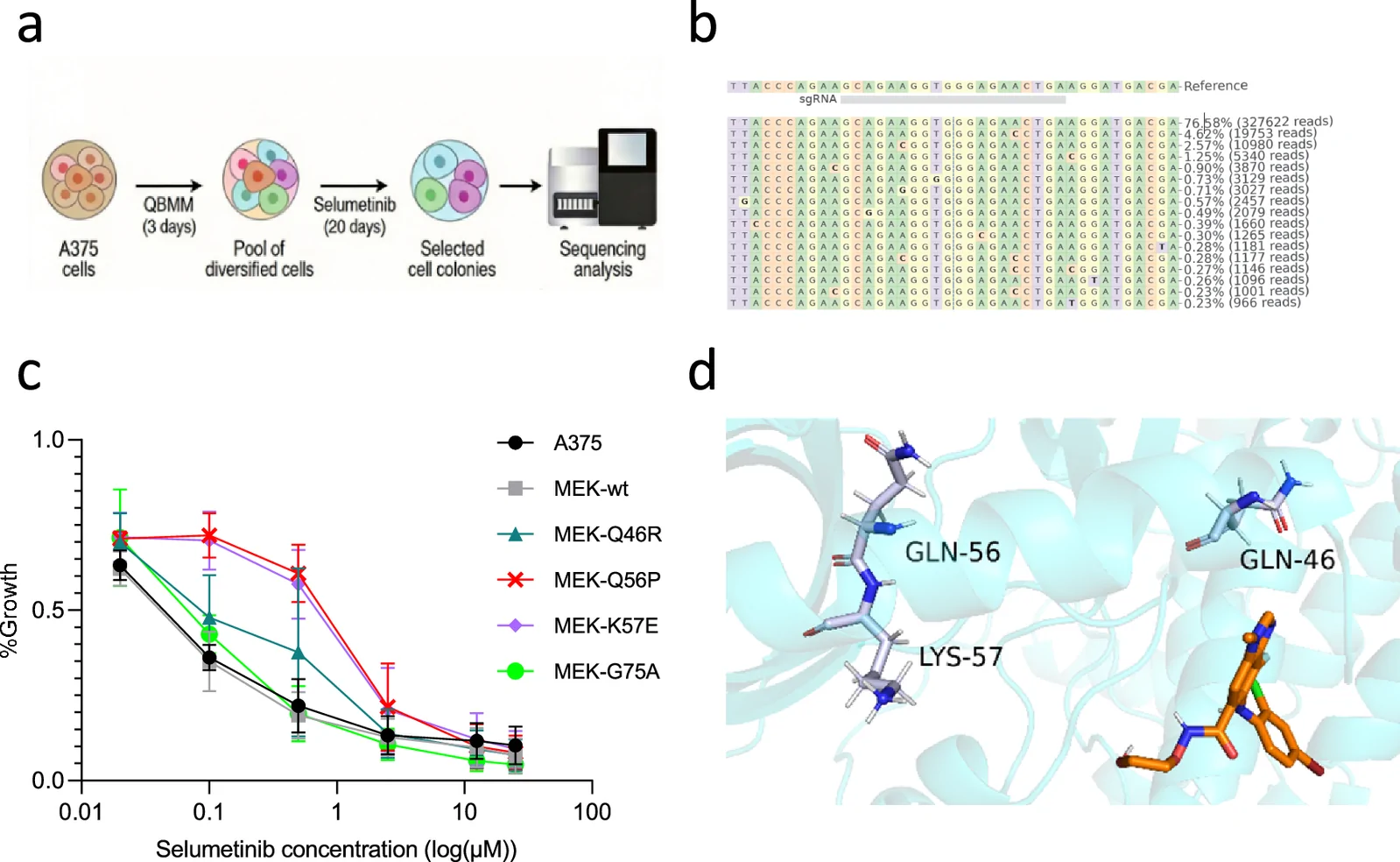
Quadruple base-mutating module (QBMM) enables the identification of MEK1 inhibitor-resistance mutations in the endogenous genome. **a** Workflow of the QBMM MEK1 inhibitor–resistance screen. A375 cells were transfected with QBMM and diversified for 3 days. The genomically diversified cells were then selected with selumetinib for 20 days. Genomes of resistant clones were harvested and subjected to deep sequencing. **b** Schematic showing the distribution of sequencing reads across part of MEK1 exon 2. **c** Growth inhibition curves of parental A375 cells and A375 cells expressing either wild-type MEK1 or the indicated possible MEK1 inhibitor-resistant alleles. Data are presented as mean ±  s.d. from three biological replicates. **d** Structure of MEK1 in complex with selumetinib (PDB code: 7JUZ). MEK1 is depicted in blue. Selumetinib is shown in orange. Source data are provided as a Source Data file.

We sought to apply QBMM to perform functional mutagenesis in endogenous coding sequences. We initially screened for mutations within *MEK1* (also known as *MAP2K1*) that promote resistance to small-molecule drug inhibition. MEK inhibitors target the MAPK-ERK (extracellular signal-regulated kinase) pathway, which is aberrantly upregulated in one-third of all cancers^23^. We used QBMM to diversify exons of the *MEK1* gene in A375 cells, with deep sequencing confirming a saturated mutational spectrum (Supplementary Fig. 17), a melanoma line sensitive to MEK inhibition. After 3 days of mutagenesis, we selected cells for resistance to selumetinib (Fig. 6a). We targeted exons 2, 3, and 4, which contain previously identified mutation hotspots^24,25^. Nine gRNAs targeting *MEK1* exon were designed to introduce saturation mutagenesis, and exons with high mutation frequencies were identified by deep sequencing (Fig. 6b). We recovered drug-tolerant mutants and identified MEK1 mutations by targeted amplicon sequencing (see Methods). We identified four candidate mutations (Q46R, Q56P, K57E, and G75A) (Table 1). Comparison of pre- and post-selection allele frequencies showed marked enrichment of all four identified variants (Supplementary Fig. 18).

**Table 1:** Table of candidate hits identified in the MEK1 inhibitor-resistance screen

CDS base position	Reference base	Alternate base	Phenotype	Allele frequency (%) in culture 1	Allele frequency (%) in culture 2
137	A	G	Q46R		19.6%
167	A	C	Q56P	21.3%	
169	A	G	K57E	46.6%	50.3%
223	C	G	G75A	47.5%	

To validate the candidates, we constructed five expression plasmids: one expressing wild‑type MEK1 and four expressing MEK1 with each of the individual mutations. The ability of these mutations to confer selumetinib resistance was then evaluated using growth inhibition assays (Fig. 6d). A375 cells expressing wild‑type MEK1 (MEK‑wt) or MEK1 G75A exhibited GI₅₀ values comparable to that of parental A375 cells (≈29 nM; Fig. 6d). In contrast, MEK1 Q46R, Q56P, and K57E increased selumetinib GI₅₀ values by 3.8‑fold, 40.9‑fold, and 35.1‑fold respectively. Notably, the Q56P mutation has previously been reported to confer selumetinib resistance^24^, and all three mutations (Q46R, Q56P, and K57E) have also been shown to confer resistance to the clinically relevant MEK inhibitor trametinib^25^, further supporting their functional significance as MEK1 hotspot resistance alleles. G75A likely represents a concurrent passenger mutation, as it was co‑detected with K57E in several cDNA clones.

Structural analysis revealed that all three resistance‑conferring mutations cluster in or near the N‑terminal helix A of MEK1, a well‑characterized autoinhibitory element that normally restrains kinase activity by packing against the core kinase domain (Fig. 6e). Q56P introduces a helix‑breaking proline residue, thereby disrupting the helical conformation of helix A. K57E reverses the charge of a key lysine in the negative regulatory domain^26^, which likely weakens the autoinhibitory interaction. Q46R, located immediately upstream of helix A, introduces a bulky, positively charged side chain that likely perturbs the N-terminal autoinhibitory segment. Consequently, all three mutations relieve the autoinhibitory constraint, leading to elevated basal MEK1 kinase activity and sustained ERK signaling even in the presence of allosteric MEK inhibitors, thereby conferring strong (Q56P, K57E) or moderate (Q46R) resistance. Overall, these results demonstrate that, by generating an unbiased mutational spectrum, QBMM enables the identification of drug resistance-conferring mutations.

## Discussion

The development of versatile base editors capable of conducting all possible base conversion not only expands the genome-editing toolbox for basic research but also holds significant therapeutic potential for treating diseases caused by genetic mutations. In this study, we engineered a compact dual base editor A&GBE with a single MPG domain, which could simultaneously mediate A-to-Y and G-to-Y editing. This strategy eliminated the need for separate MPG3.0^5^ and MPG6.3^6^ constructs. This design not only simplifies delivery but also facilitates the integration of additional effector proteins for broader mutagenesis applications.

To enhance the editing precision of AYBE and A&GBE, we refined their adenine-editing window by replacing the TadA8e adenine deaminase with our previously evolved TadA variants (E4/E14/E11-TadA)^22^, which constrains deamination activity to a narrower range. Notably, the guanine editing activity of A&GBE substituted with E14-TadA was improved slightly compared with A&GBE (Fig. 2b). We propose that the reduced DNA-binding affinity of these TadA mutants—attributed primarily to charge-neutralizing or reversing substitutions (e.g., N38D, R39G, R107G)—may alleviate steric hindrance with MPG, thereby facilitating the substrate engagement of MPG. This observation is consistent with earlier reports indicating that TadA (V106W) and nCas9 compete for DNA access^21^. Our results demonstrate a practical strategy for constructing functional fusion proteins without compromising the activity of individual components.

Existing targeted hyper mutators in mammalian cells are constrained by a narrow mutational spectrum, primarily yielding C-to-T or G-to-A substitutions^19,20^. Recent advances, including T7 polymerase-driven continuous editing (TRACE^23^/TRIDENT^24^) and helicase-assisted continuous editing (HACE^27^), have extended the editable range to over 1000–2000 bp. However, these systems remain limited to C-to-T or A-to-G substitutions mediated by AID or TadA respectively. In contrast, our strategy—which could combines multiplexed gRNAs—enables long-range, all-four-base diversification in endogenous mammalian genomes. QBMM therefore provides unique opportunities for disease analysis. For instance, it can recapitulate multi‑nucleotide variants found in cancer (e.g., concurrent mutations in oncogenes and tumor suppressors) or in neurological disorders such as Charcot–Marie–Tooth disease. It also enables saturation mutagenesis to functionally annotate variants of uncertain significance in disease‑linked genes, such as *MYH7* in cardiomyopathies. These capabilities bridge unbiased hypermutation with clinically relevant disease analysis. We anticipate that QBMM will deliver key advances in high-resolution sequence-function mapping, systematic analysis of genetic regulation and disease, and efficient directed evolution in mammalian cells.

## Methods

### Construct design

Lentiviral plasmid expressing AYBE (Lv-AYBE-Cas9n-Puro) and GYBE (Lv-Cas9n-MPGv6.3_Puro) were made by cloning AYBE and GYBE gene sequences from AYBEv3 (Addgene, #193967) and gGBEv6.3-SpCas9 (Addgene, #202629) respectively. Lv-AGBE-Puro was constructed through the exchange of MPGv3.0 with MPGv6.3 gene in gGBEv6.3-SpCas9 plasmid (Addgene, #202629).

DNA ligation was performed using ClonExpress® II One Step Cloning Kit (Vazyme), and all clones were transformed into home-made Stbl3 Competent *E. Coli*. Individual colony was grown at 37 °C (non-lentiviral plasmid) or 30 °C (lentiviral plasmid) at 220 rpm for ~16–22 h. Plasmids were purified with HiPure Plasmid Mini/midi Kit, HiPure Plasmid EF Midi Kit (Magen, #P1002-02, #P1003-02, and #P1113-02). All constructs were validated by Sanger sequencing, and sequence alignment was conducted with CodonCode Aligner.

### Cell culture

A375 melanoma cells (donated by Dr. Jing Yang, Gannan Medical University) and HEK293T cells (purchased from National Collection of Authenticated Cell Cultures) were grown in a humidified 37 °C incubator with 5% CO_2_ using DMEM media supplemented with 100 I.U./mL penicillin and 100 µg/mL streptomycin (Thermo Fisher, #15140163).

### Transfection

Transfection experiment for functional validation: HEK293T cells were seeded into 24-well plates and transfected with 1500 ng BE and 500 ng gRNA plasmids per well using EZtrans (Life iLAB, China) following the manufacturer’s instructions. Protospacer sequences for all mammalian genomic sites targeted in this study are provided in Supplementary Data 1. Twenty-four hours after transfection, the culture media were exchanged with fresh culture media supplemented with 2 µg/ml puromycin (Solarbio, China) and 20 µg/ml blasticidin (Solarbio, China). 3 days after transfection, genomic DNA was extracted for PCR amplification, and the resultant PCR products were subjected to deep sequencing.

### Genomic DNA extraction and PCR amplification

Genomic DNA extraction for base editing test assay: The harvested cells were mixed with 30 μl lysis buffer (10 mM Tris-HCl (pH 8.0), 50 mM KCl, 1.5 mM MgCl_2_, 0.5% Nonidet P-40, 0.5% Tween−20, and 100 μg/ml proteinase K (Roche)) and incubated under the following procedure: 68 °C for 30 min, 16 °C for 2 min and 98 °C for 5 min. The lysates were centrifuged at 12,000 rpm for 3 min and the supernatant was collected as template. The target genomic loci were amplified from genomic DNA using Phanta® Max Super-Fidelity DNA Polymerase (Vazyme) using the following program: 95 °C for 5 min, 35 cycles of 95 °C for 30 s, 56 °C for 30 s, 72 °C for 20 s, and 72 °C for 5 min. The purified PCR products were subjected to Sanger sequencing or targeted deep sequencing. Sanger sequencing data were analyzed by EditR for base-editing activity.

### Target sequencing of endogenous sites and data analysis

Endogenous target sites of interest were amplified from genomic DNA as previously described^9^. Genomic DNA was extracted, and the regions of interest in the target sites were amplified by PCR using site-specific primers (Supplementary Data 2). In brief, the amplicons were ligated to adapters, and sequencing was performed on the Illumina platforms. The targeted amplicon sequencing reads were processed using fastp with default parameters, and further amplicon sequencing analyses were performed by CRISPResso (Supplementary Data 3).

### Targeted deep sequencing and data analysis

PCR products were purified by the VAHTS DNA clean Beads (Vazyme) and then subjected to library construction and high-throughput sequencing on an Illumina Nova 6000 with PE150 mode (Annoroad, Beijing, China). The numbers of sequencing reads obtained for each amplicon are provided in Supplementary Data 4. The amplicon sequencing data were analyzed using CRISPResso2 (v.2.0.3) in the batch mode, with parameters “--q 30 --base_edit --wc -8 --fastq_output --base_editor_output --write_cleaned_report --place_report_in_output_folder.” Editing efficiency was quantified from the “Quantification_window_nucleotide_percentage_table.txt” table. Indels were quantified from the “Alleles_frequency_table_around_sgRNA_*.txt” table. The results including A-to-Y conversion rates, C-to-non-Y (A-to-G) conversion rates, G-to-Y conversion rates, G-to-non-Y(G-to-A) and indel rates were calculated. Sum of base substitution frequency were calculated with the formula: Sum of N Nucleotide substitution feq.= sum (N mutated to other bases) of specific treatment-sum (N mutated to other bases) of untreated cells.

### MEK1 inhibitor-resistance screen

A375 cells grown in the absence of antibiotics were seeded into 6-well plates (Biofil, China). At 12–14 h post-seeding, cells at approximately 70% confluency were transfected with 12 μL of Lipofectamine 2000 (Thermo Fisher Scientific) and 4.5 μg of dTAGBE+AID plasmids, 1.5 μg of MS2-sgRNAs-AID targeting MEK1 exons 2, 3, and 4, following the manufacturer’s instructions. Three days later, cells were split into three wells of a 6-well plate and selected with selumetinib (TargetMol, Cat#6218) at 100 nM for 20 days. A portion of preselection cells was harvested as a control. Cells were passaged every 3 days to maintain confluency below 70%. After selection, the total RNA was extracted from cells using an RNAsimple Total RNA Kit (Tiangen Biotech, Beijing, China, Cat#DP419) according to the manufacturer’s instructions. The cDNA was synthesized by reverse transcription using HiScript II 1st Strand cDNA Synthesis Kit (Vazyme). The MEK1 was amplified by forward primer CGACGGCTCTGCAGTTAAC and reverse primer GTCTGACTGCACAGAGTAATG. Sequencing libraries for cDNA were generated using the S-1.2× TN5 library enrichment Mix. Libraries were subsequently sequenced using the SaluSeq Nimbo sequencing kit on the SaluSeq Nimbo sequencer.

### MEK1-mutations expression constructs

The full-length MEK1 coding sequence was amplified from cDNA of the post-selection cells under the following program: 95 °C for 5 min, 30 cycles of 95 °C for 30 s, 56 °C for 30 s, 72 °C for 20 s, and 72 °C for 5 min. The purified PCR products were ligated into an expression plasmid using ClonExpress® II One Step Cloning Kit (Vazyme). Plasmids were purified with HiPure Plasmid EF Midi Kit (Magen, cat#P1113-02). All constructs were validated by Sanger sequencing, and sequence alignment was conducted with CodonCode Aligner.

### Cell viability and growth inhibition assays

Cell viability was assessed using an Enhanced CCK-8 Cell Counting Kit (Genesand, CCK102-500) according to the manufacturer’s protocol. In brief, ~3 × 10^5^ A375 cells in 2.5 ml of growth medium were seeded in a six-well plate. The cells were transfected with 6 μg of the respective MEK1 expression plasmids. One day after transfection, cells were transferred to 48‑well plates and cultured in 250 μL of medium containing 5% FBS and various concentrations of selumetinib. After 7 days, cells were washed with DPBS and incubated with 200 μL medium containing CCK‑8 reagent for 1.5 hours. Afterward, 100 μL cell culture medium was replaced in the 96-well plate for measuring at 450 nm to assess cell viability. Cell viability was then calculated by comparing the absorbance of treated samples to that of control (untreated) samples, typically using the formula: Percentage of cell viability =  (Atreatment − Ablank)/(Acontrol − Ablank) × 100%. Final values can then be obtained by averaging results from triple biology replicates for plotting.

### Statistics and reproducibility

GraphPad Prism 9 software was used to analyze the data. All numerical values are presented as mean ± s.d, as noted otherwise. Data from the targeted deep sequencing were corrected for between-session variation using Factor Correction. No statistical method was used to predetermine sample size. No data were excluded from the analyses. The experiments were not randomized. The Investigators were not blinded to allocation during experiments and outcome assessment.

### Reporting summary

Further information on research design is available in the Nature Portfolio Reporting Summary linked to this article.

## Supplementary information

Supplementary Information

Description of Additional Supplementary Files

Supplementary Data 1

Supplementary Data 2

Supplementary Data 3

Supplementary Data 4

Reporting Summary

Transparent Peer Review file

## Source data

Source Data

## Data Availability

The raw high-throughput sequencing data generated in this study have been deposited in the NCBI Sequence Read Archive (SRA) database under PRJNA1237726. The protein structure shown in Fig. 6e was retrieved from the Protein Data Bank (PDB ID: 7JUZ). Source data for all figures and annotated plasmid sequences are provided as Source Data file. Source data are provided with this paper.
