# Supplementary material for: A dual A-to-Y and G-to-Y base editor and a four-base concurrent hypermutator in mammalian cells
Source: Nat Commun. 2026 Aug 27;17:10292. doi: 10.1038/s41467-026-77241-5 (PMC13619609; doi:10.1038/s41467-026-77241-5)
Supplement: Supplementary file 1 [file 41467_2026_77241_MOESM1_ESM.pdf]

Supplementary information

Supplementary Fig. 1. Editing frequencies at NAN and NGN motifs.

Supplementary Fig. 2. Cumulative substitution frequencies across the protospacer.

Supplementary Fig. 3. Frequencies of A→T/C/G and G→T/C/A editing at genomic sites targeted by GYBE, AYBE, A&GBE, and ABE8e.

Supplementary Fig. 4. Indel frequencies in HEK293T cells at 12 genomic sites after treatment with GYBE, AYBE, A&GBE, and ABE8e.

Supplementary Fig. 5. AYBE and A&GBE, with previously evolved adenine deaminase, exhibit narrowed editing windows.

Supplementary Fig. 6. Indel frequencies in HEK293T cells at 12 genomic sites.

Supplementary Fig. 7. Comparison of AYBE, GYBE and A&GBE editing based on Cas9n and dCas9.

Supplementary Fig. 8. Cumulative substitution frequencies across the protospacer.

Supplementary Fig. 9. Indel frequencies in HEK293T cells at 6 genomic sites after treatment with GYBE, dGYBE, AYBE, dAYBE, A&GBE, and d A&GBE.

Supplementary Fig. 10. Schematic showing the sequencing reads of S13-targeting region.

Supplementary Fig. 11. Schematic workflow for the experiments in Figs. 4 and 5.

Supplementary Fig. 12. Distribution of base substitution frequencies along the GFP sequence.

Supplementary Fig. 13. Proportions of A, C, G, and T mutated to other bases for each group.

Supplementary Fig. 14. Distribution of base substitution frequencies along the GFP sequence.

Supplementary Fig. 15. Proportions of A, C, G, and T mutated to other bases for each group.

Supplementary Fig. 16. Comparison of cell viability among base editors at 72 h.

Supplementary Fig. 17 | Distribution of base substitution frequencies along MEK1 exons 2, 3, and 4.

Supplementary Fig. 18 | Enrichment of MEK1 mutations following selumetinib selection.

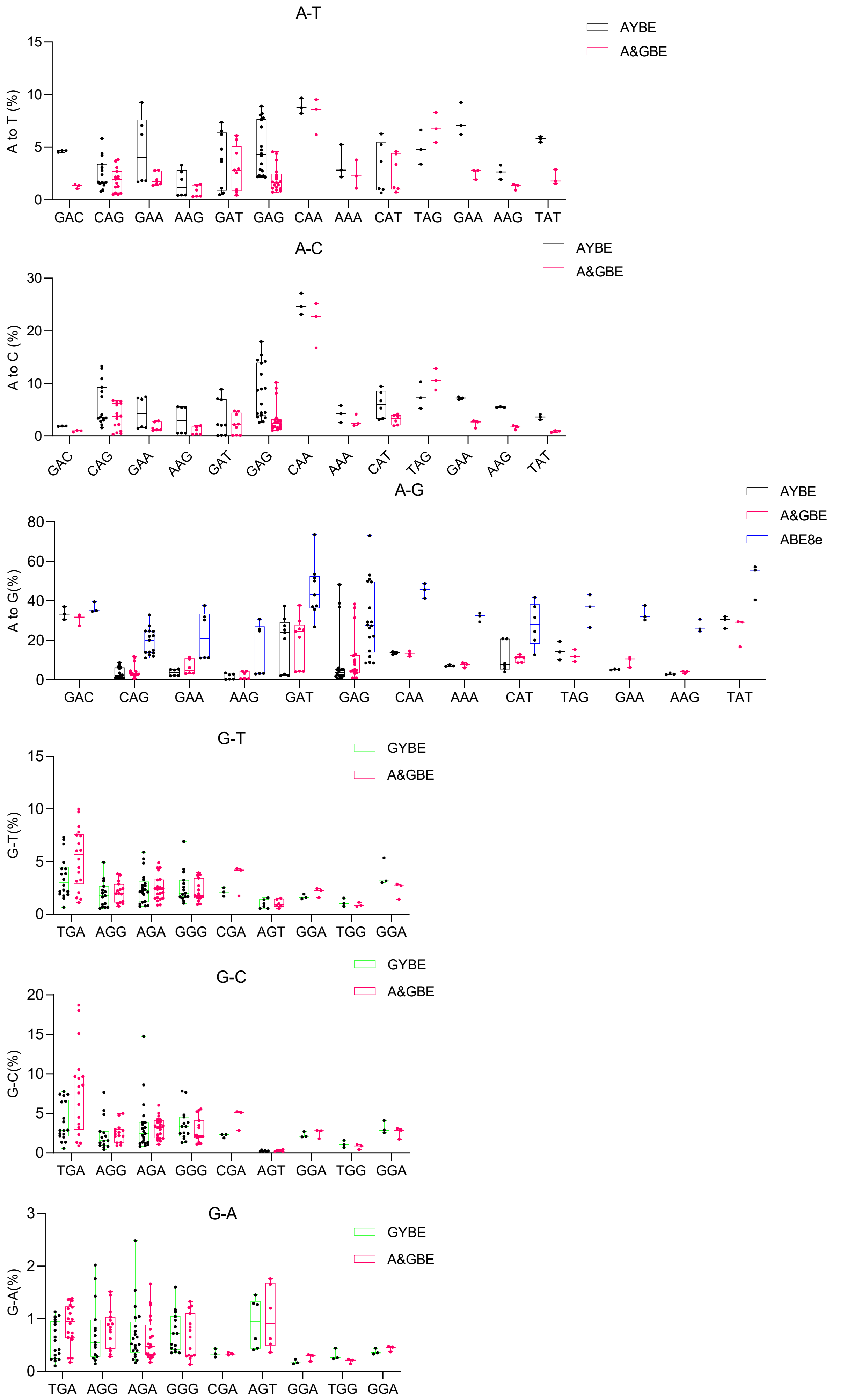

Supplementary Fig. 1 | Editing frequencies at NAN and NGN motifs. Frequencies of A-to-T/C/G and G-to-T/C/A editing at NAN or NGN DNA motifs within the protospacer positions (5–11 for A, 3–12 for G) at the genomic sites targeted by GYBE, AYBE, A&GBE, and ABE8e in Fig. 1 are shown; each dot represents the editing frequency at a motif from the corresponding site. Source data are provided as a Source Data file.

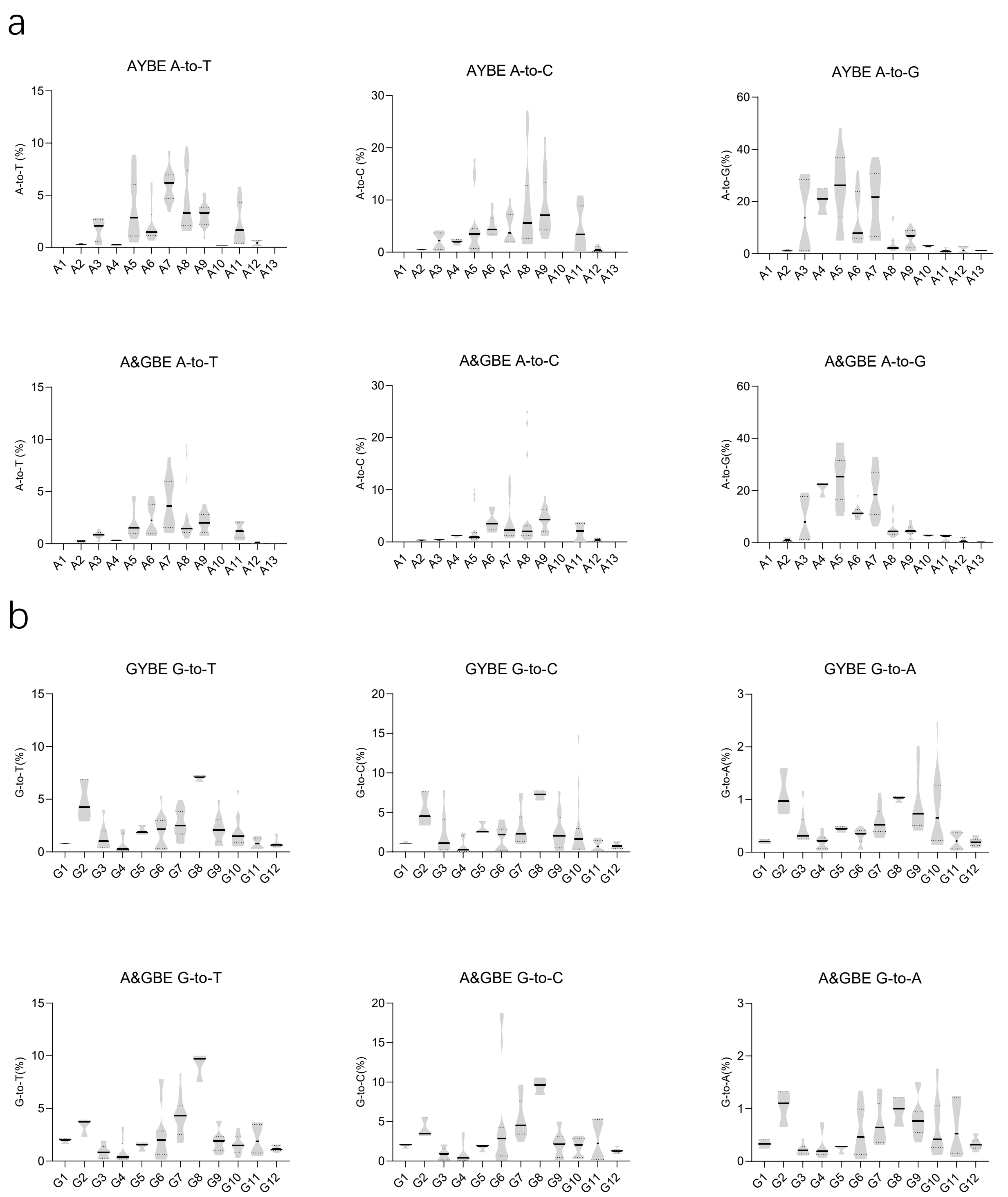

Supplementary Fig. 2 | Cumulative substitution frequencies across the protospacer. (a) Frequencies of A→T/C/G and (b) G→T/C/A substitutions at the edited sites with PAM at positions 21–23. Data are based on Fig. 1. Source data are provided as a Source Data file.

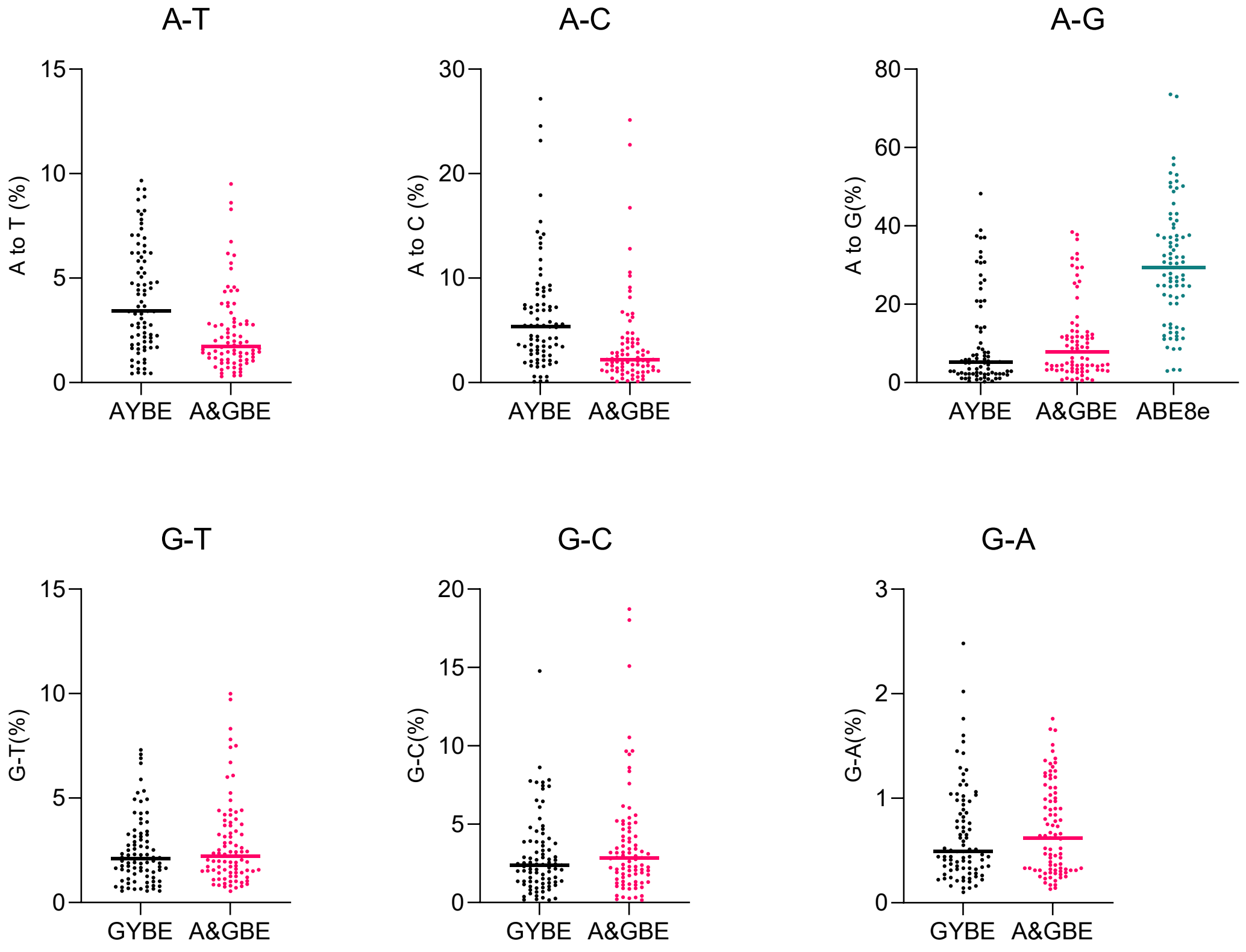

Supplementary Fig. 3 | Frequencies of A→T/C/G and G→T/C/A editing at genomic sites targeted by GYBE, AYBE, A&GBE, and ABE8e. Each dot represents the editing frequency. The figures were generated based on data from Fig. 1. Source data are provided as a Source Data file.

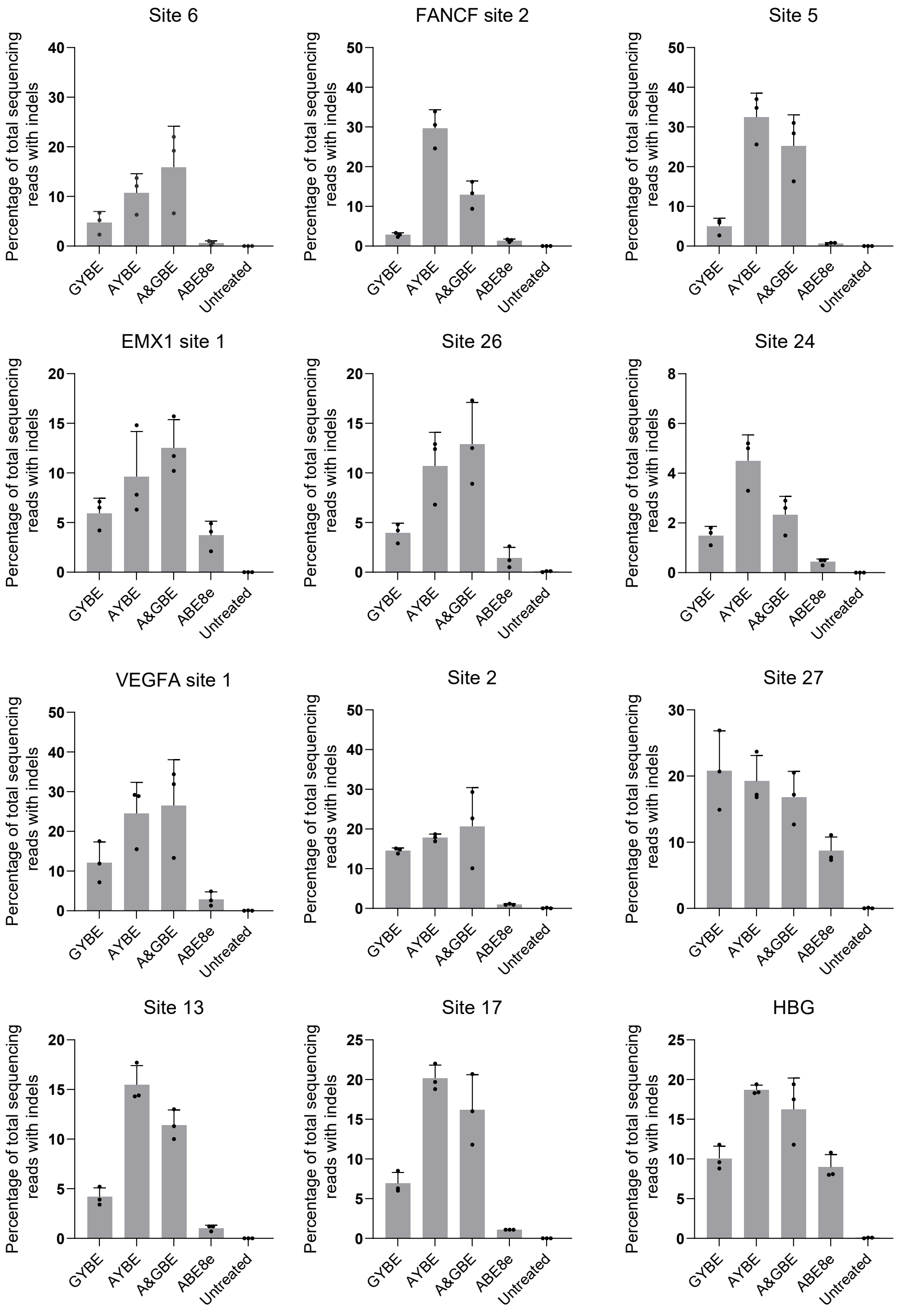

Supplementary Fig. 4 | Indel frequencies in HEK293T cells at 12 genomic sites after treatment with GYBE, AYBE, A&GBE, and ABE8e. Indel frequencies at each of the 12 genomic sites. Data were derived from Fig. 1. Source data are provided as a Source Data file.

a

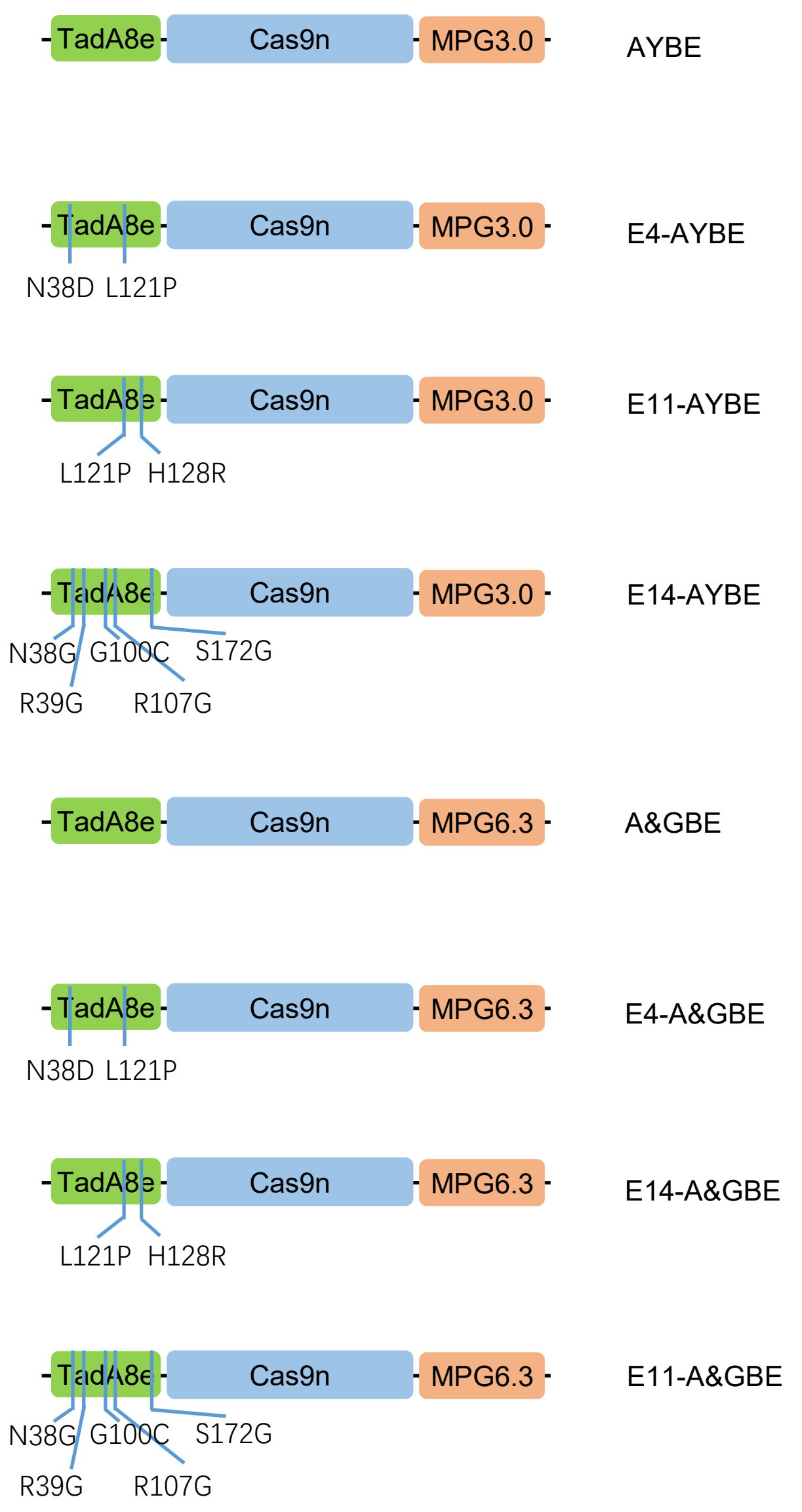

TadA8e
Cas9n
MPG3.0
AYBE
TadA8e
Cas9n
MPG3.0
N38D L121P
E4-AYBE
TadA8e
Cas9n
MPG3.0
L121P H128R
E11-AYBE
TadA8e
Cas9n
MPG3.0
N38G G100C S172G
R39G R107G
E14-AYBE
TadA8e
Cas9n
MPG6.3
A&GBE
TadA8e
Cas9n
MPG6.3
N38D L121P
E4-A&GBE
TadA8e
Cas9n
MPG6.3
L121P H128R
E14-A&GBE
TadA8e
Cas9n
MPG6.3
N38G G100C S172G
R39G R107G
E11-A&GBE

b

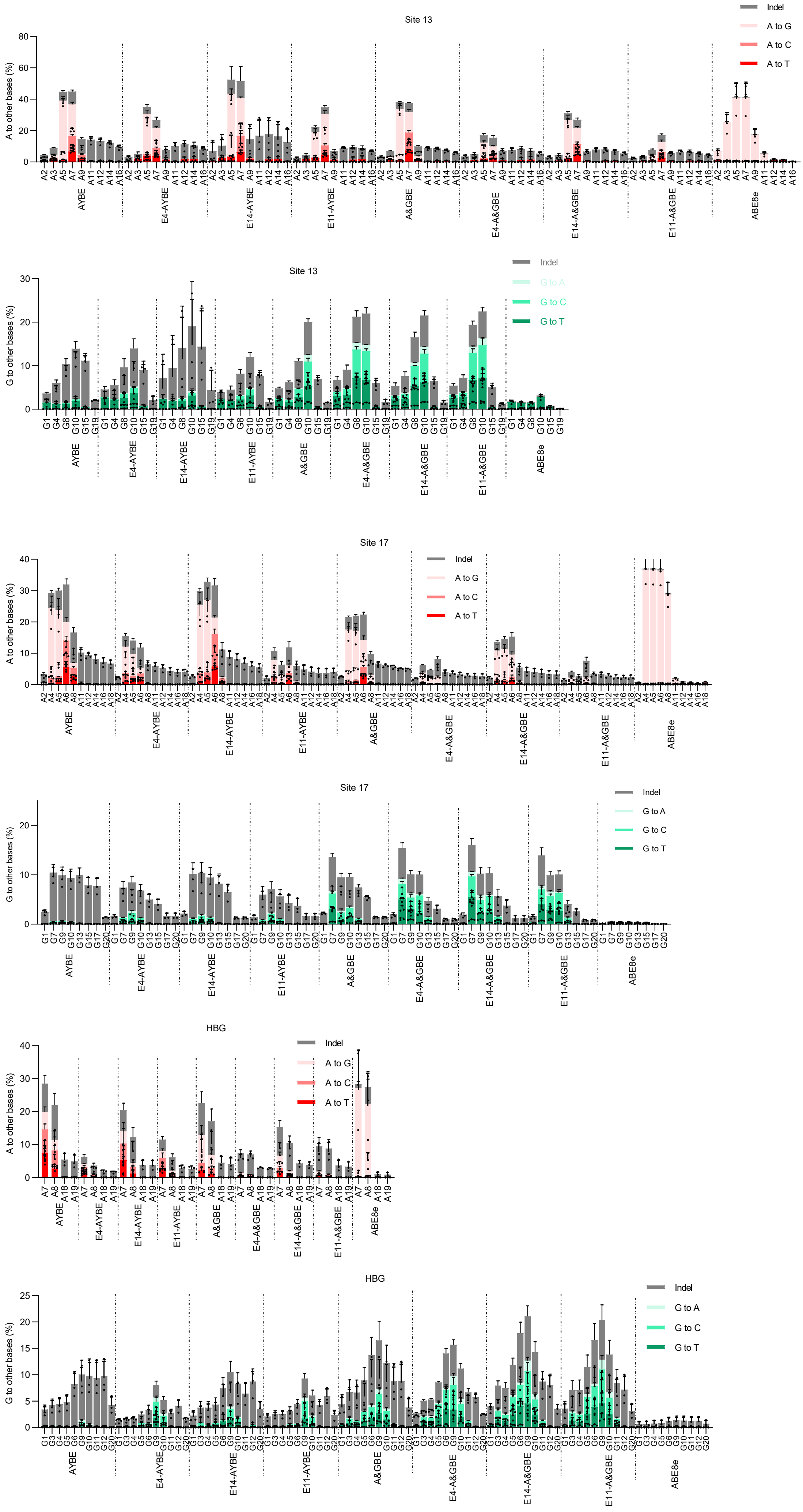
Site 13
A to other bases (%)
Indel
A to G
A to C
A to T
AYBE
E4-AYBE
E14-AYBE
E11-AYBE
A&GBE
E4-A&GBE
E14-A&GBE
E11-A&GBE
ABE8e
Site 13
G to other bases (%)
Indel
G to A
G to C
G to T
Site 17
A to other bases (%)
Indel
A to G
A to C
A to T
Site 17
G to other bases (%)
Indel
G to A
G to C
G to T
HBG
A to other bases (%)
Indel
A to G
A to C
A to T
HBG
G to other bases (%)
Indel
G to A
G to C
G to T

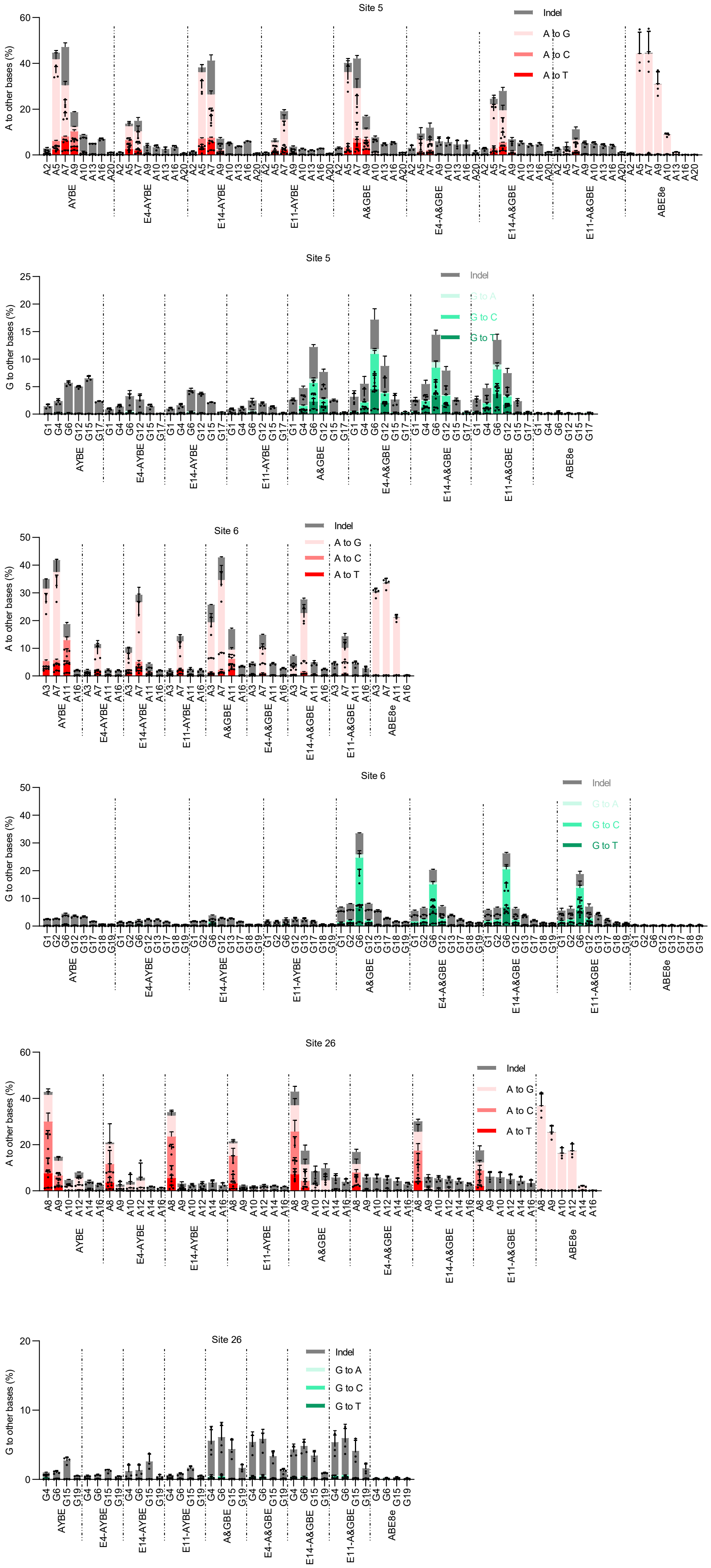

Site 5
A to other bases (%)
Indel
A to G
A to C
A to T
AYBE
E4-AYBE
E14-AYBE
E11-AYBE
A&GBE
E4-A&GBE
E14-A&GBE
E11-A&GBE
ABE8e
Site 5
G to other bases (%)
Indel
G to A
G to C
G to T
Site 6
A to other bases (%)
Indel
A to G
A to C
A to T
Site 6
G to other bases (%)
Indel
G to A
G to C
G to T
Site 26
A to other bases (%)
Indel
A to G
A to C
A to T
Site 26
G to other bases (%)
Indel
G to A
G to C
G to T

C

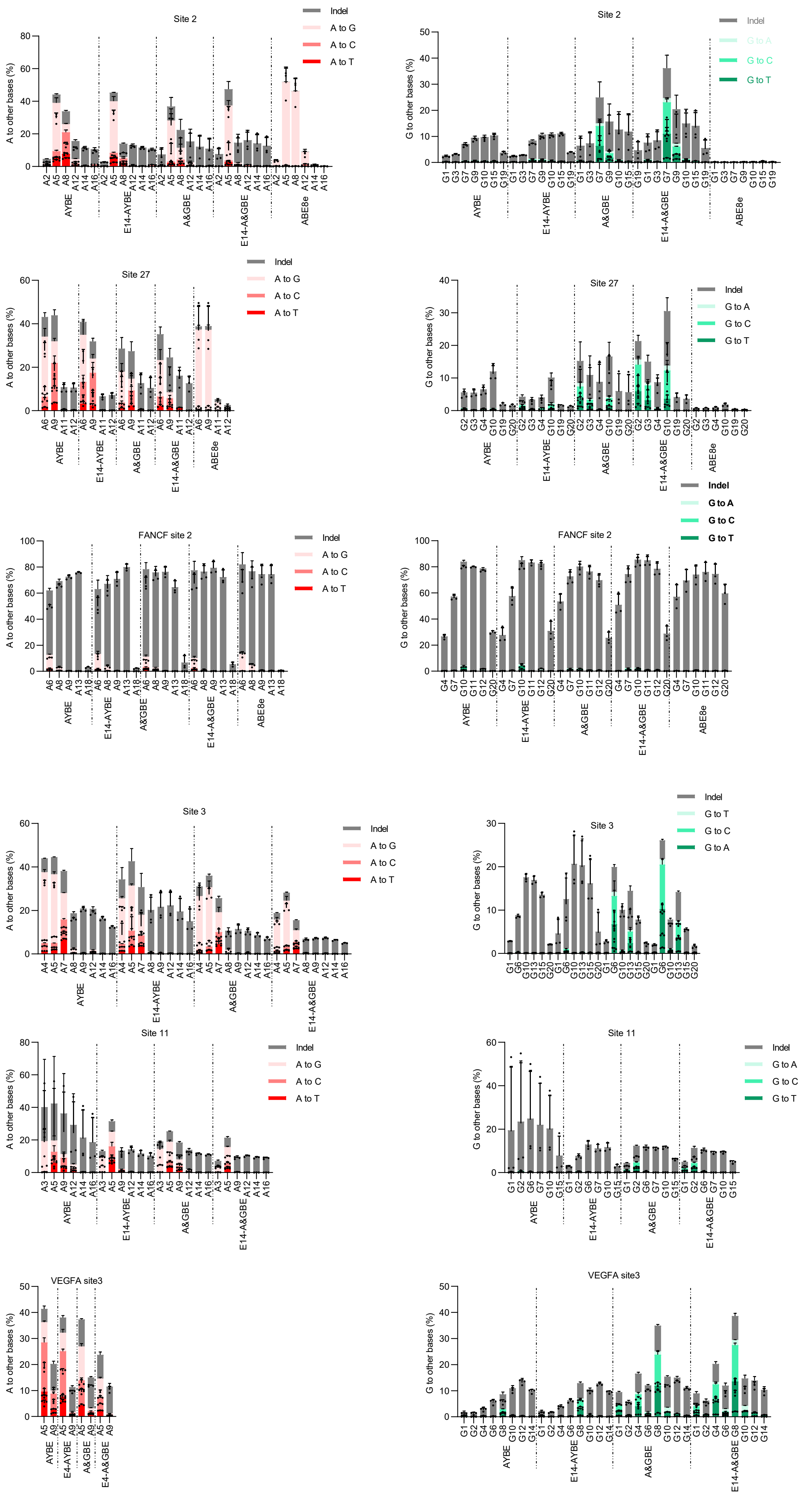
Site 2
A to other bases (%)
G to other bases (%)
Indel
A to G
A to C
A to T
G to A
G to C
G to T
AYBE
E14-AYBE
A&GBE
E14-A&GBE
ABE8e
Site 27
FANCF site 2
Site 3
Site 11
VEGFA site3
E4-AYBE
E4-A&GBE

Supplementary Fig. 5 | AYBE and A&GBE, with previously evolved adenine deaminase, exhibit narrowed editing windows. a, Schematics of AYBE, E4-AYBE, E14-AYBE, E11-AYBE, A&GBE, E4-A&GBE, E14-A&GBE, and E11-A&GBE. b, Editing profiles of AYBE, E4-AYBE, E14-AYBE, E11-AYBE, A&GBE, E4-A&GBE, E14-A&GBE, and E11-A&GBE at six genomic sites. c, Editing profiles of AYBE, E14-AYBE, A&GBE, and E14-A&GBE at six genomic sites. Values and error bars represent the mean and s.d. from three biological replicates performed on separate days. Source data are provided as a Source Data file.

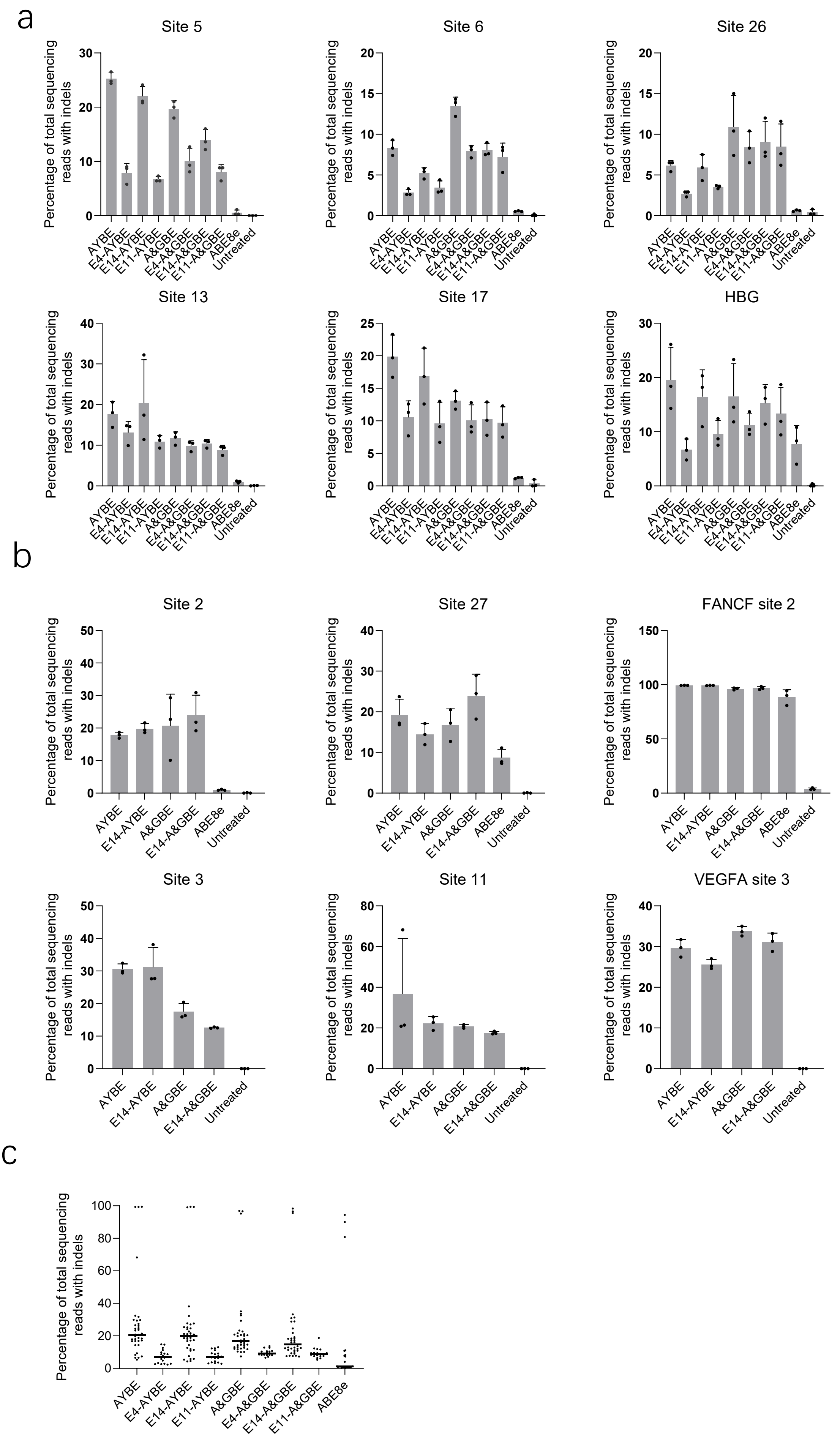

Supplementary Fig. 6 | Indel frequencies in HEK293T cells at 12 genomic sites. a, Indel frequencies after treatment with AYBE, E4-AYBE, E14-AYBE, E11-AYBE, A&GBE, E4-A&GBE , E14-A&GBE , E11-A&GBE and ABE8e. b, Indel frequencies after treatment with AYBE, E14-AYBE, A&GBE and E14-A&GBE. C, Cumulative indel frequencies across all sites. Data were derived from Fig. 2 and Supplementary Fig.6a,6b. Source data are provided as a Source Data file.

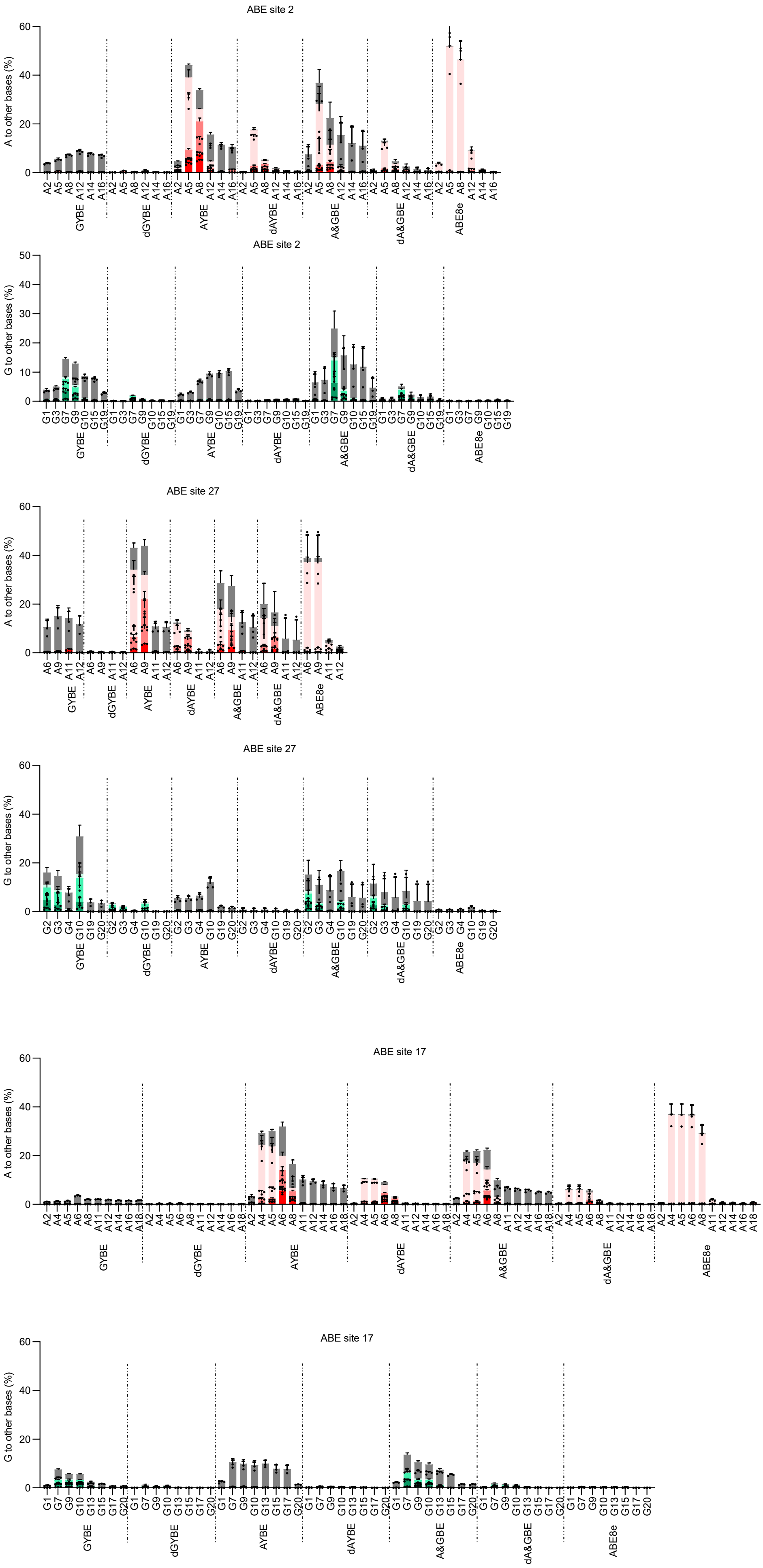
ABE site 2
A to other bases (%)
GYBE
dGYBE
AYBE
dAYBE
A&GBE
dA&GBE
ABE8e
ABE site 2
G to other bases (%)
GYBE
dGYBE
AYBE
dAYBE
A&GBE
dA&GBE
ABE8e
ABE site 27
A to other bases (%)
GYBE
dGYBE
AYBE
dAYBE
A&GBE
dA&GBE
ABE8e
ABE site 27
G to other bases (%)
GYBE
dGYBE
AYBE
dAYBE
A&GBE
dA&GBE
ABE8e
ABE site 17
A to other bases (%)
GYBE
dGYBE
AYBE
dAYBE
A&GBE
dA&GBE
ABE8e
ABE site 17
G to other bases (%)
GYBE
dGYBE
AYBE
dAYBE
A&GBE
dA&GBE
ABE8e

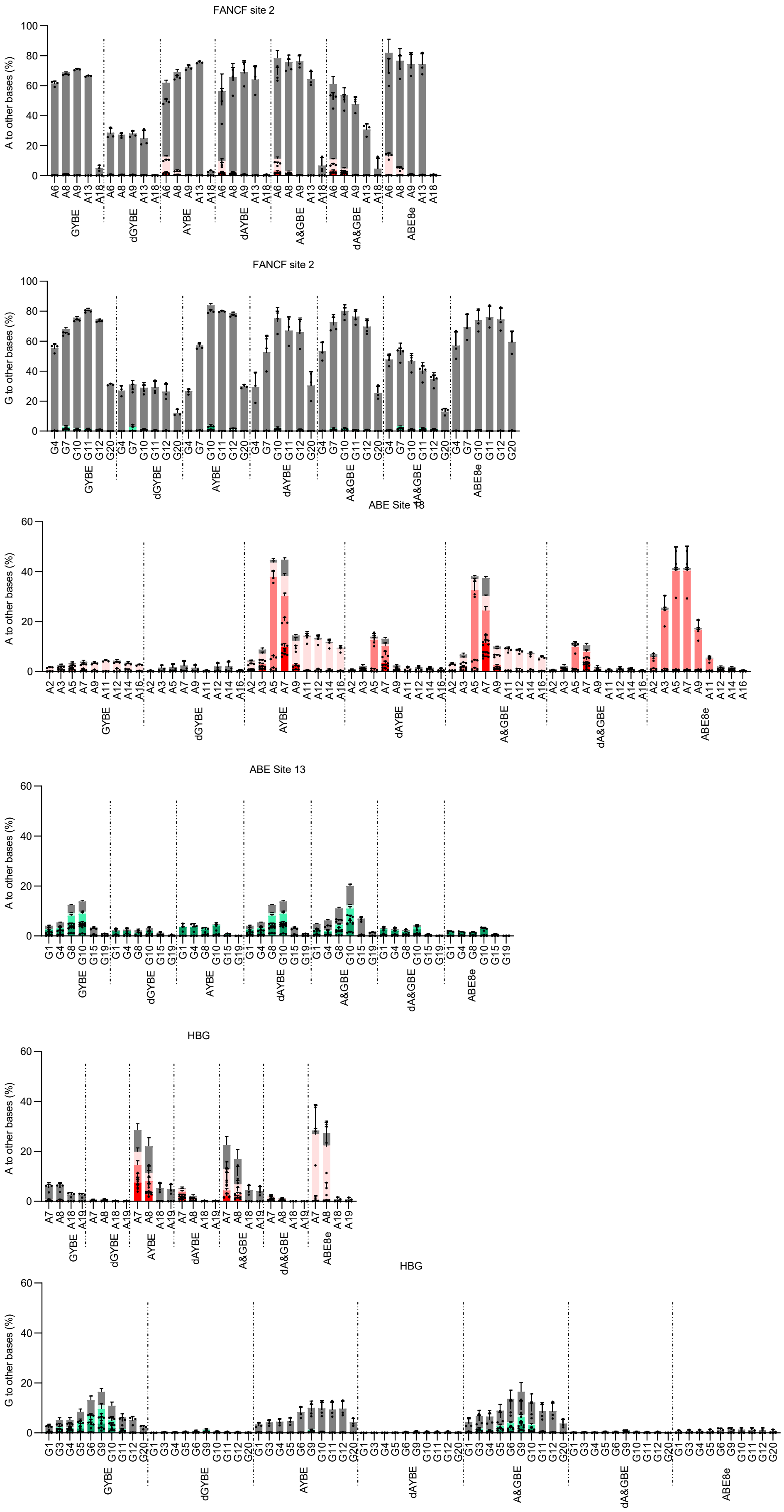

Supplementary Fig. 7 | Comparison of AYBE, GYBE and A&GBE editing based on Cas9n and dCas9. Editing of AYBE and A&GBE at six genomic sites. Values and error bars reflect the mean and s.d. of three biological replicates performed on separate days. Source data are provided as a Source Data file.

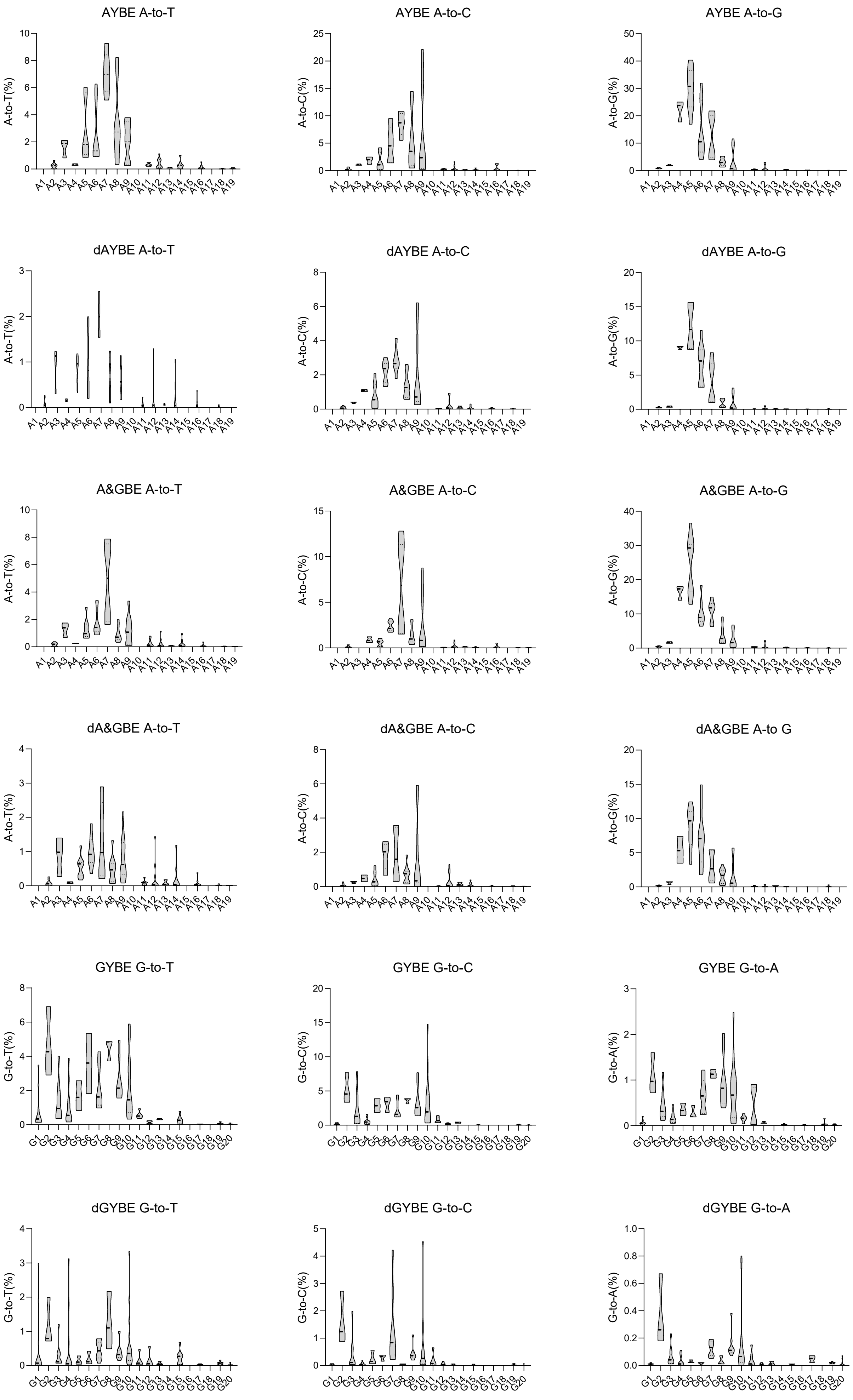

AYBE A-to-T
A-to-T(%)
AYBE A-to-C
A-to-C(%)
AYBE A-to-G
A-to-G(%)
dAYBE A-to-T
A-to-T(%)
dAYBE A-to-C
A-to-C(%)
dAYBE A-to-G
A-to-G(%)
A&GBE A-to-T
A-to-T(%)
A&GBE A-to-C
A-to-C(%)
A&GBE A-to-G
A-to-G(%)
dA&GBE A-to-T
A-to-T(%)
dA&GBE A-to-C
A-to-C(%)
dA&GBE A-to G
A-to-G(%)
GYBE G-to-T
G-to-T(%)
GYBE G-to-C
G-to-C(%)
GYBE G-to-A
G-to-A(%)
dGYBE G-to-T
G-to-T(%)
dGYBE G-to-C
G-to-C(%)
dGYBE G-to-A
G-to-A(%)

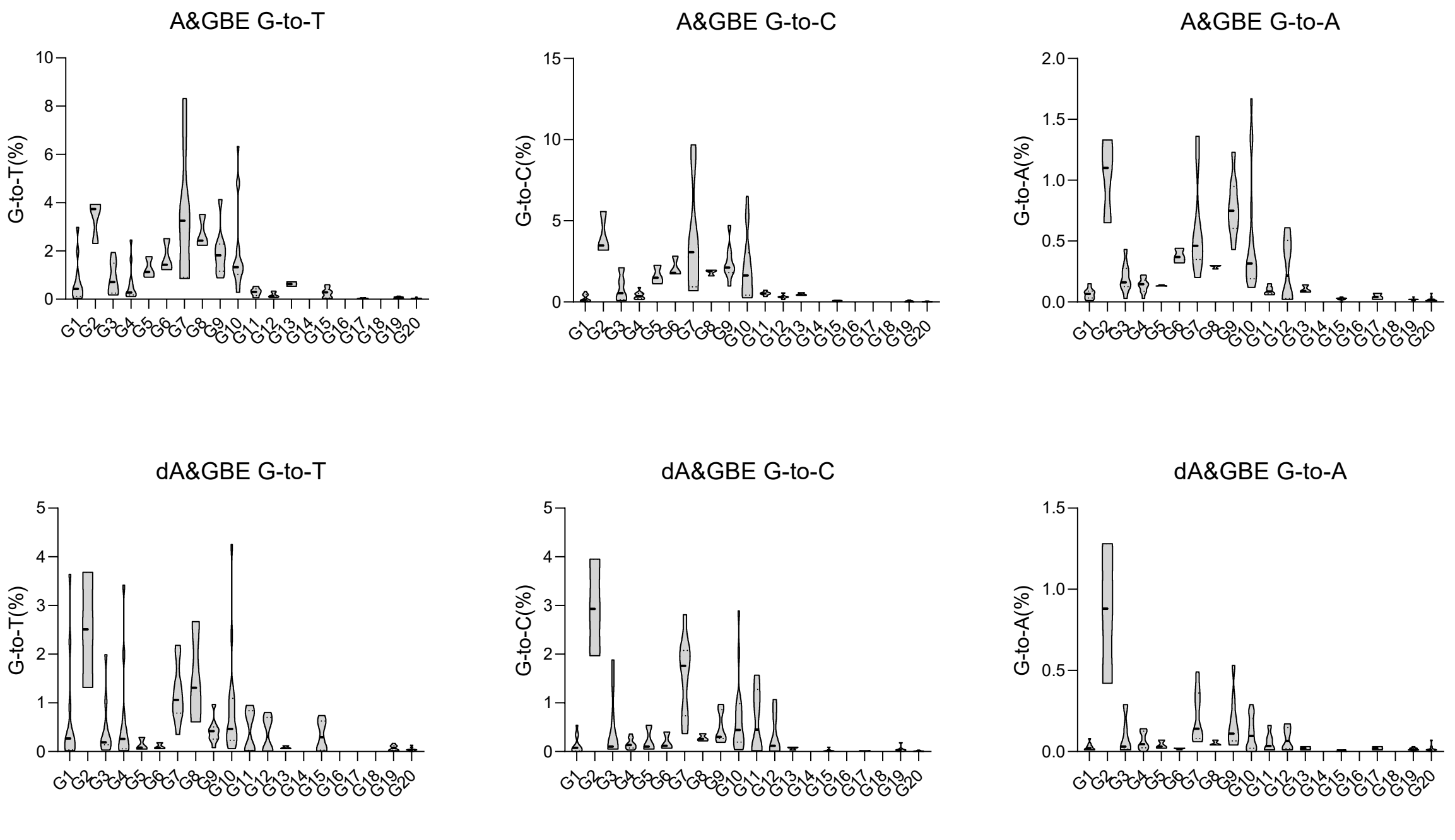

Supplementary Fig. 8 | Cumulative substitution frequencies across the protospacer. Frequencies of A→T/C/G and G→T/C/A substitutions at the edited sites (PAM at positions 21–23) are shown; data are based on Supplementary Fig. 7. Source data are provided as a Source Data file.

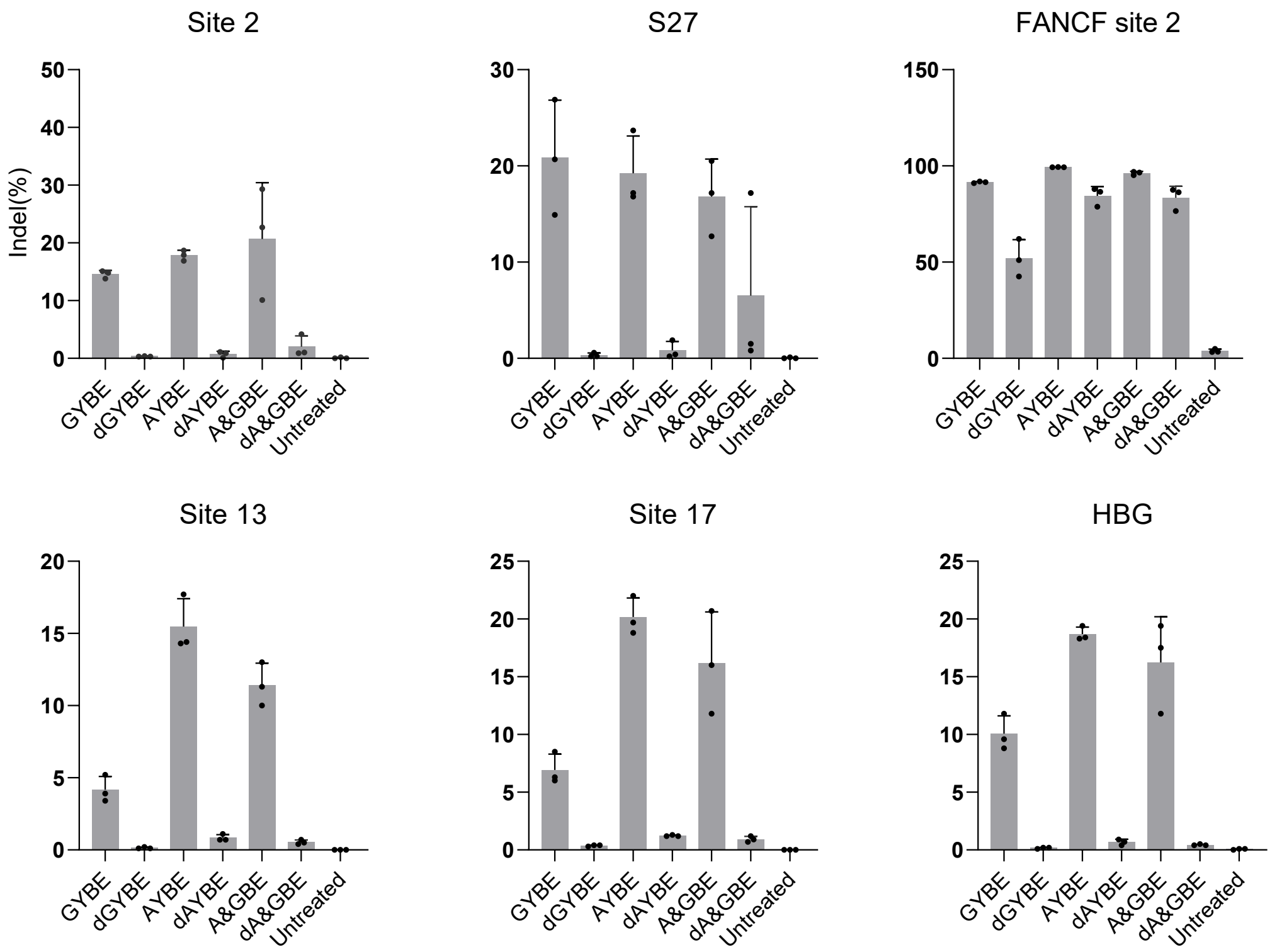

Supplementary Fig. 9 | Indel frequencies in HEK293T cells at 6 genomic sites after treatment with GYBE, dGYBE, AYBE, dAYBE, A&GBE, and d A&GBE. Indel frequencies at each of the 6 genomic sites. Data were derived from Fig. 3. Source data are provided as a Source Data file.

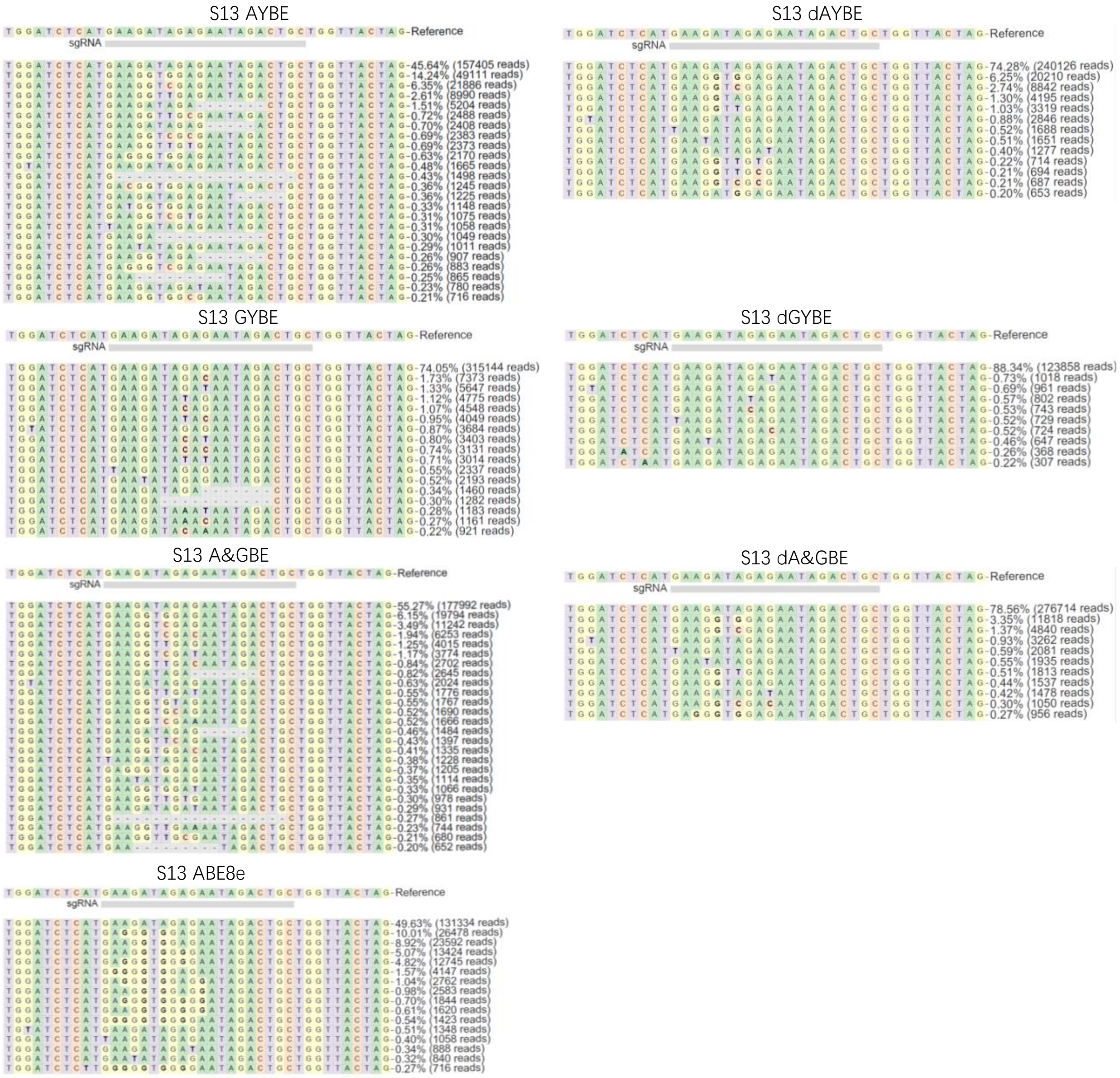

Supplementary Fig. 10 | Schematic showing the sequencing reads of S13-targeting region.

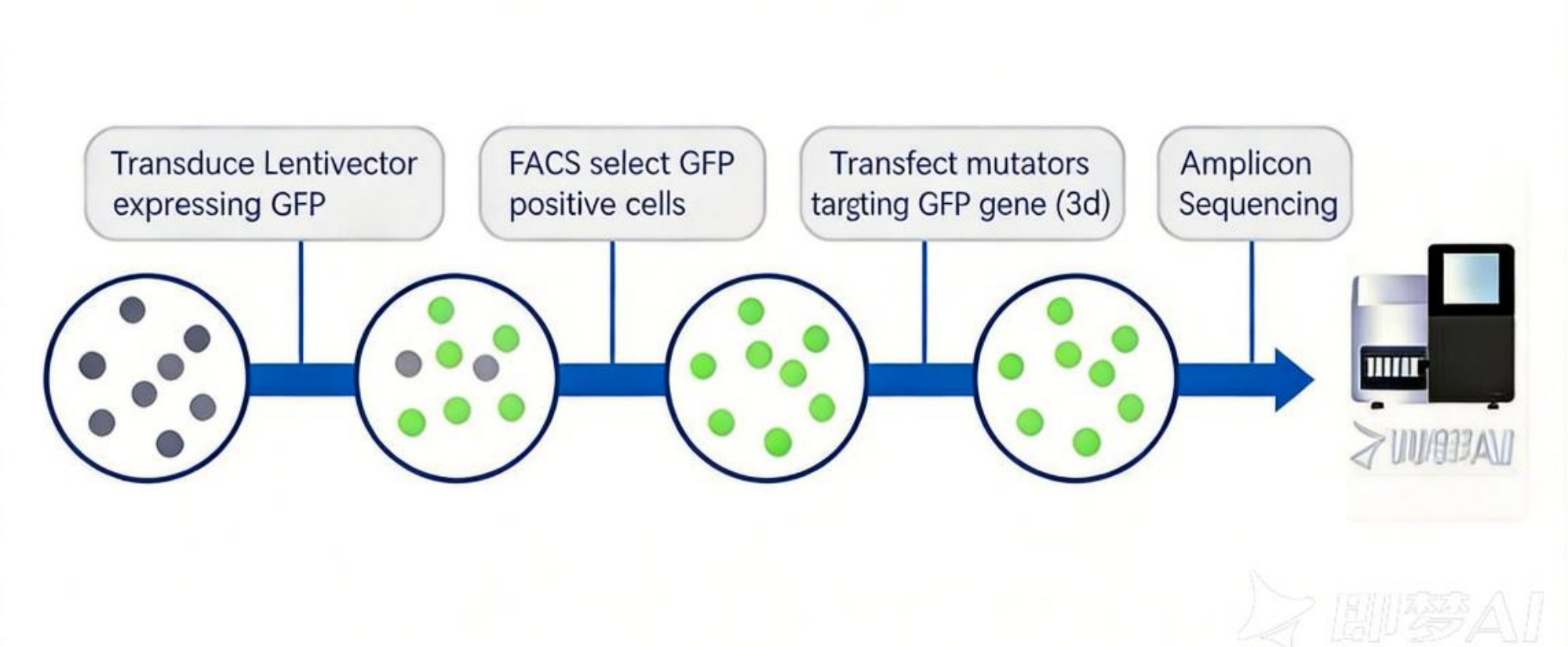

Supplementary Fig. 11 | Schematic workflow for the experiments in Figs. 4 and 5.

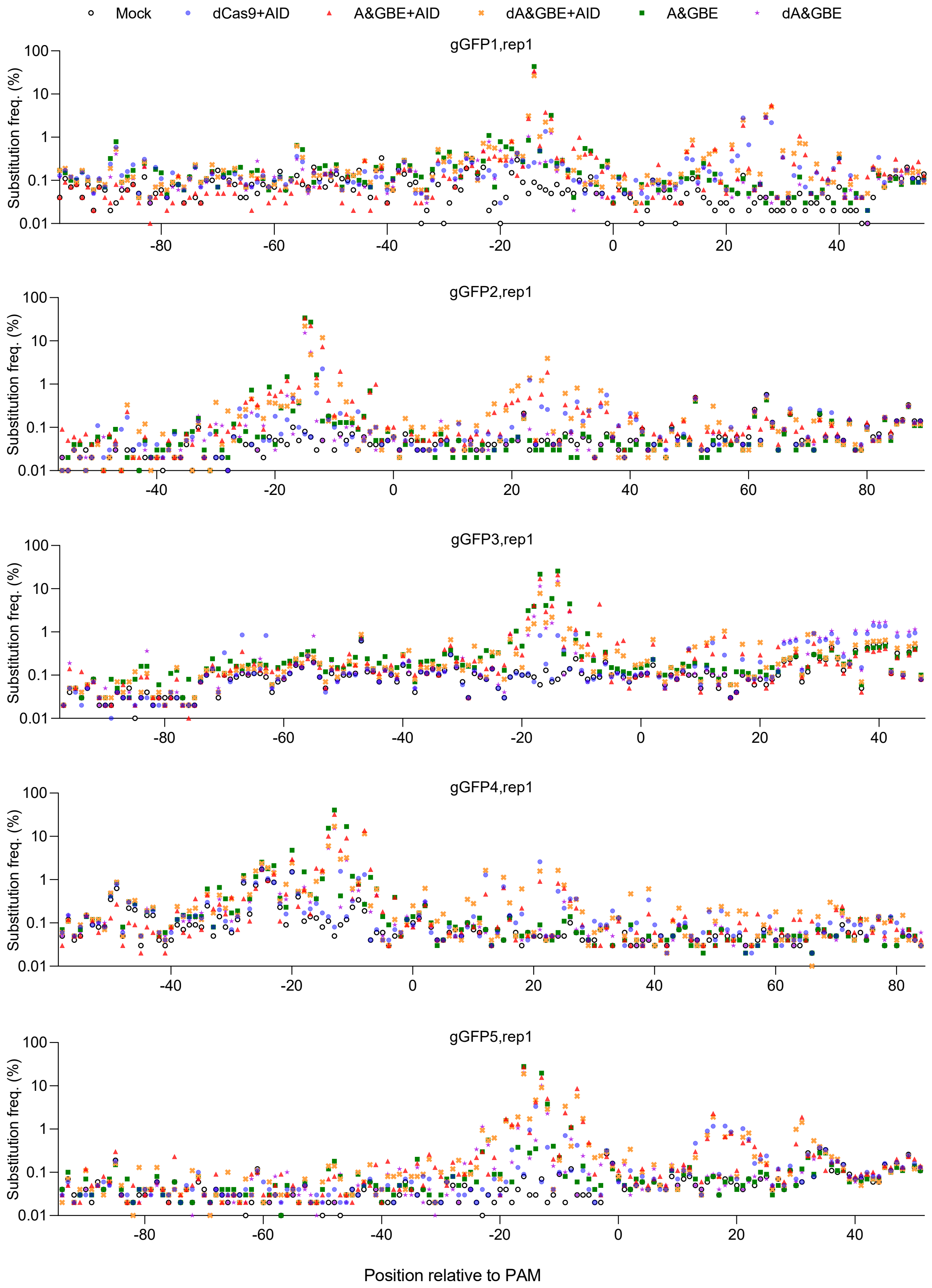
Mock
dCas9+AID
A&GBE+AID
dA&GBE+AID
A&GBE
dA&GBE
gGFP1,rep1
gGFP2,rep1
gGFP3,rep1
gGFP4,rep1
gGFP5,rep1
Substitution freq. (%)
Position relative to PAM

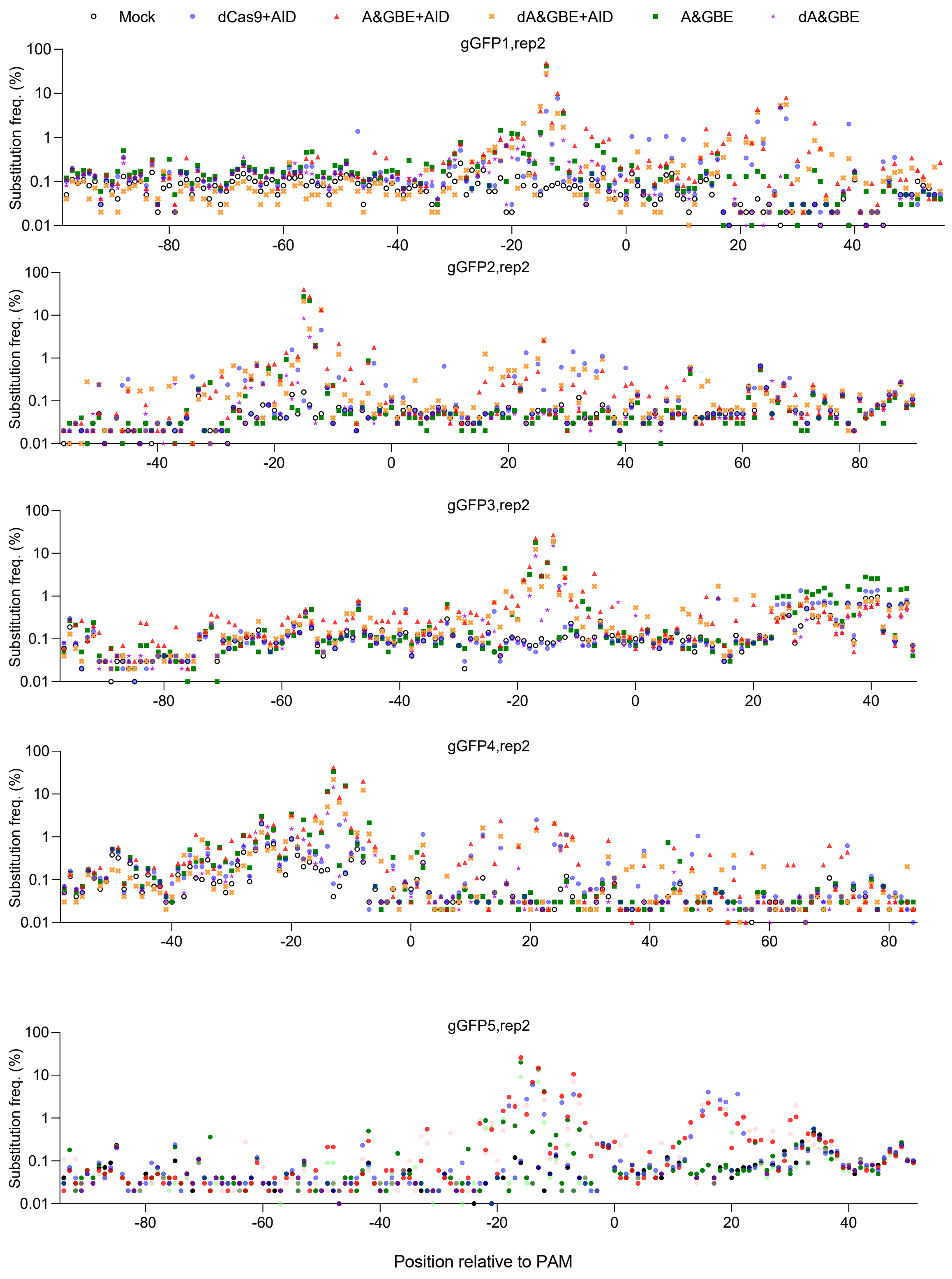

Mock
dCas9+AID
A&GBE+AID
dA&GBE+AID
A&GBE
dA&GBE
gGFP1,rep2
gGFP2,rep2
gGFP3,rep2
gGFP4,rep2
gGFP5,rep2
Substitution freq. (%)
Position relative to PAM

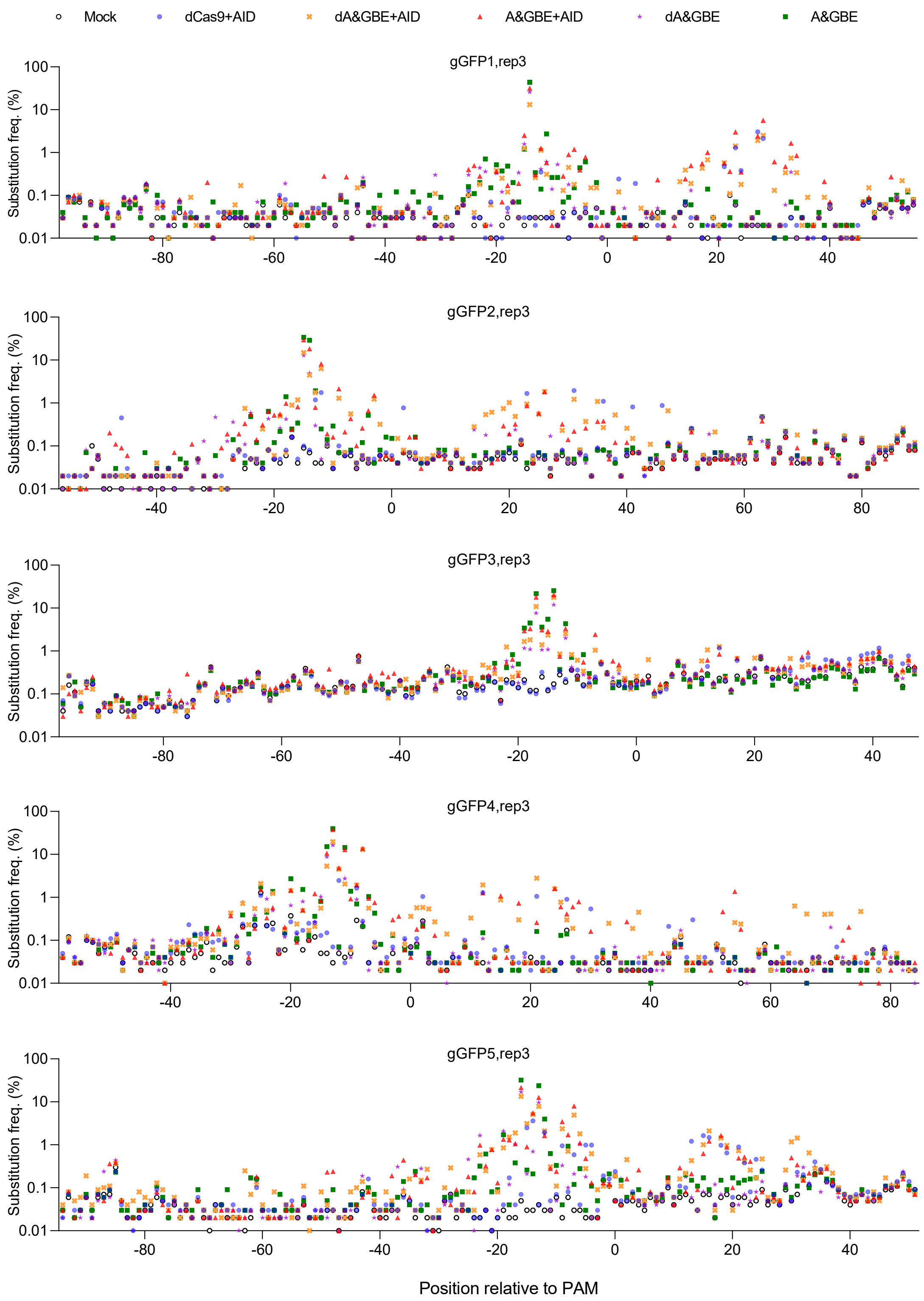

Supplementary Fig. 12 | Distribution of base substitution frequencies along the GFP sequence. Frequency (freq.) at each base was calculated as the percentage of reads containing substitutions relative to the total reads covering that base (%). Each dot represents a nucleotide. Data from three independent biological replicates are shown. Source data are provided as a Source Data file.

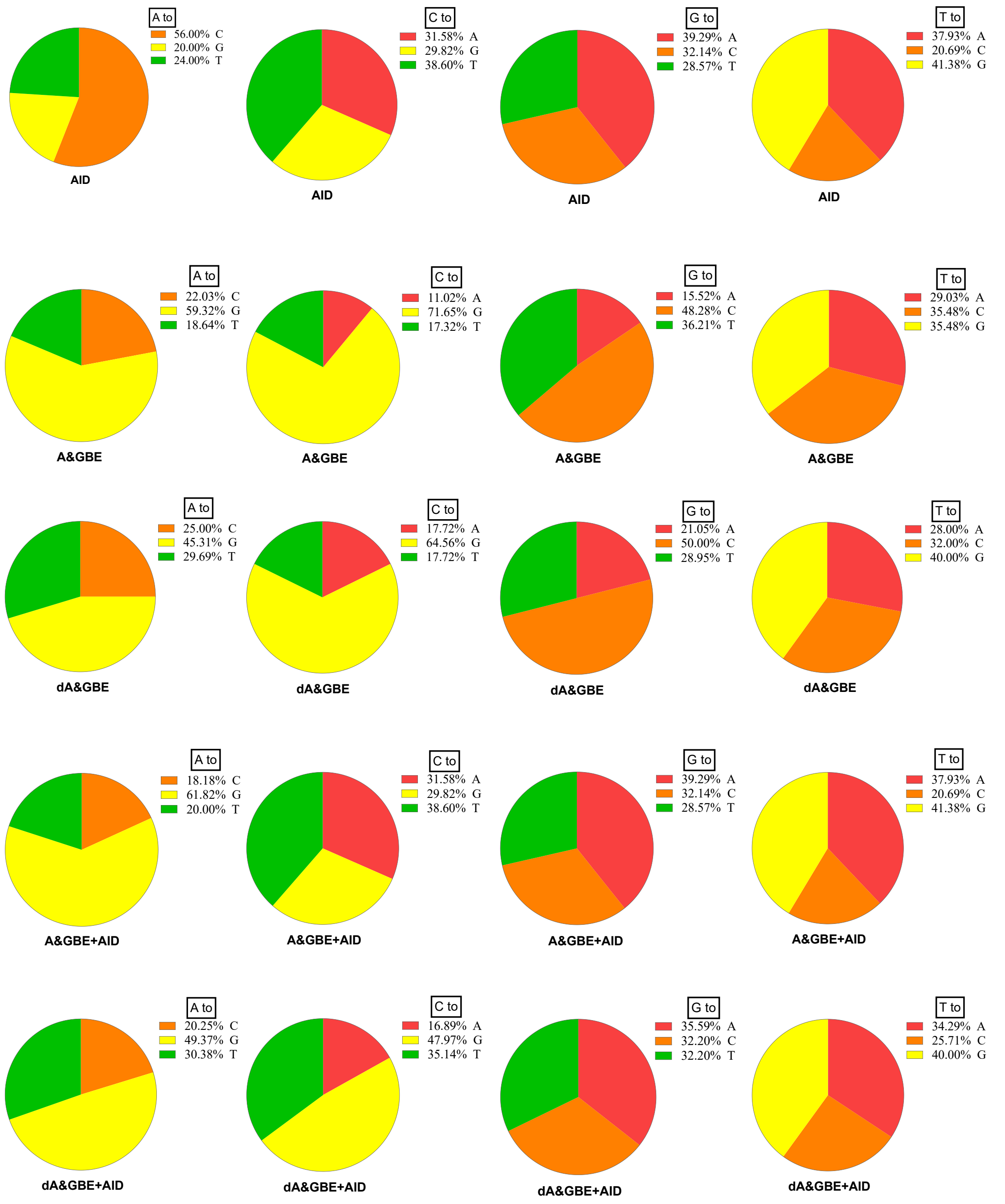

Supplementary Fig. 13 | Proportions of A, C, G, and T mutated to other bases for each group. a and b are analyzed from Fig. 4. Data are from three independent experiments. Source data are provided as a Source Data file.

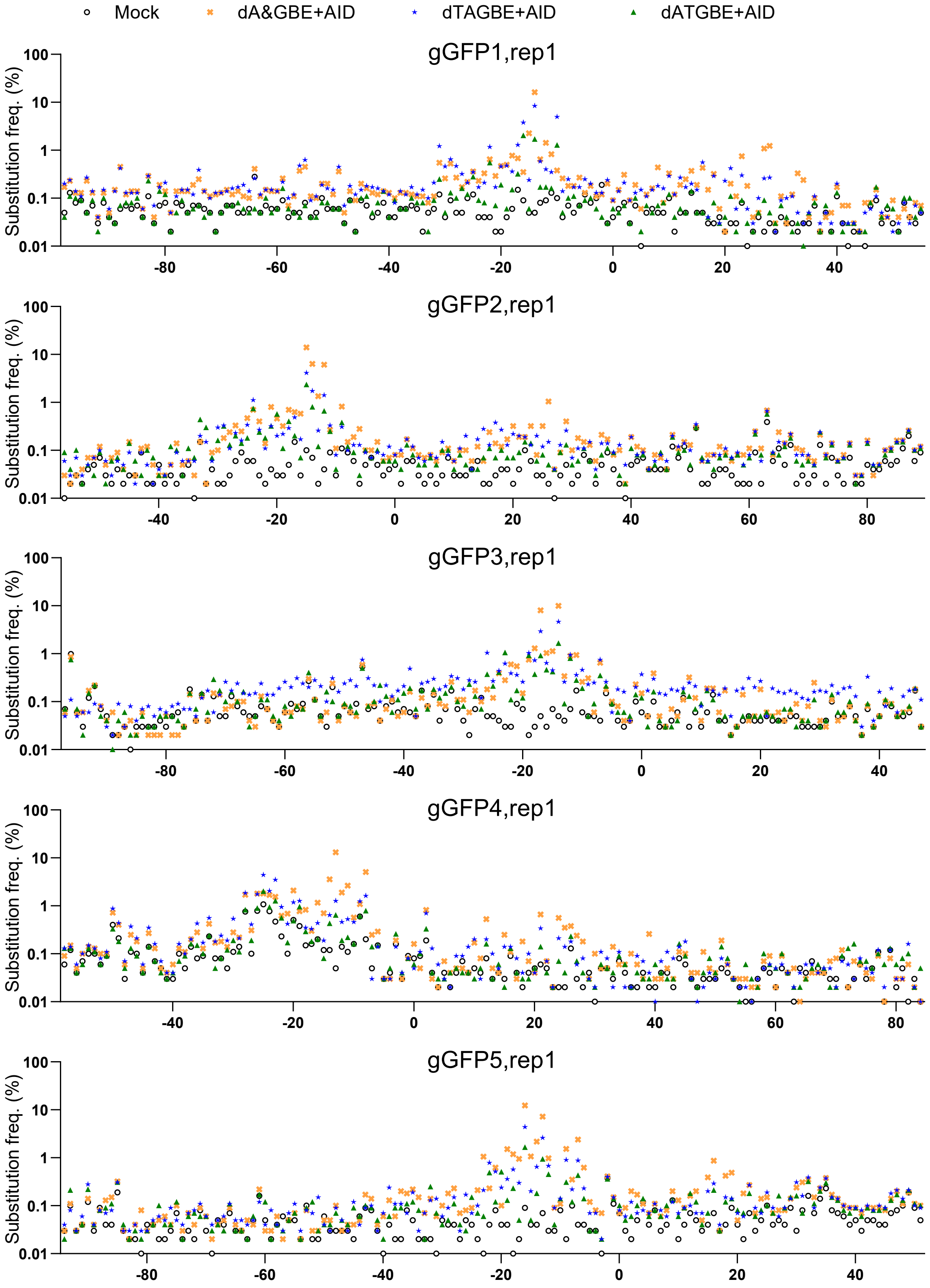
Mock
dA&GBE+AID
dTAGBE+AID
dATGBE+AID
gGFP1,rep1
gGFP2,rep1
gGFP3,rep1
gGFP4,rep1
gGFP5,rep1
Substitution freq. (%)
100
10
1
0.1
0.01
-80
-60
-40
-20
0
20
40
60
80

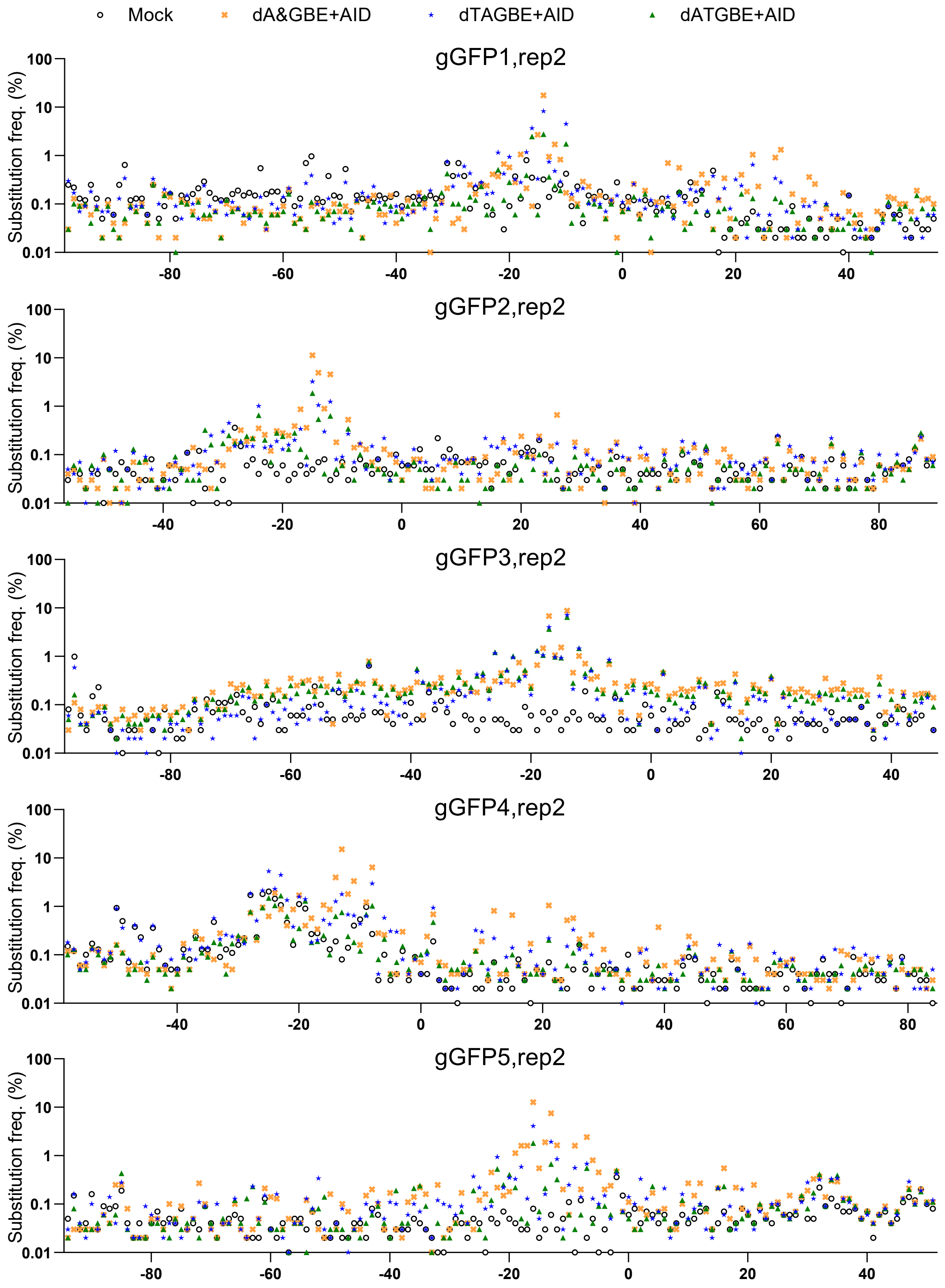

Mock
dA&GBE+AID
dTAGBE+AID
dATGBE+AID
gGFP1,rep2
gGFP2,rep2
gGFP3,rep2
gGFP4,rep2
gGFP5,rep2
Substitution freq. (%)

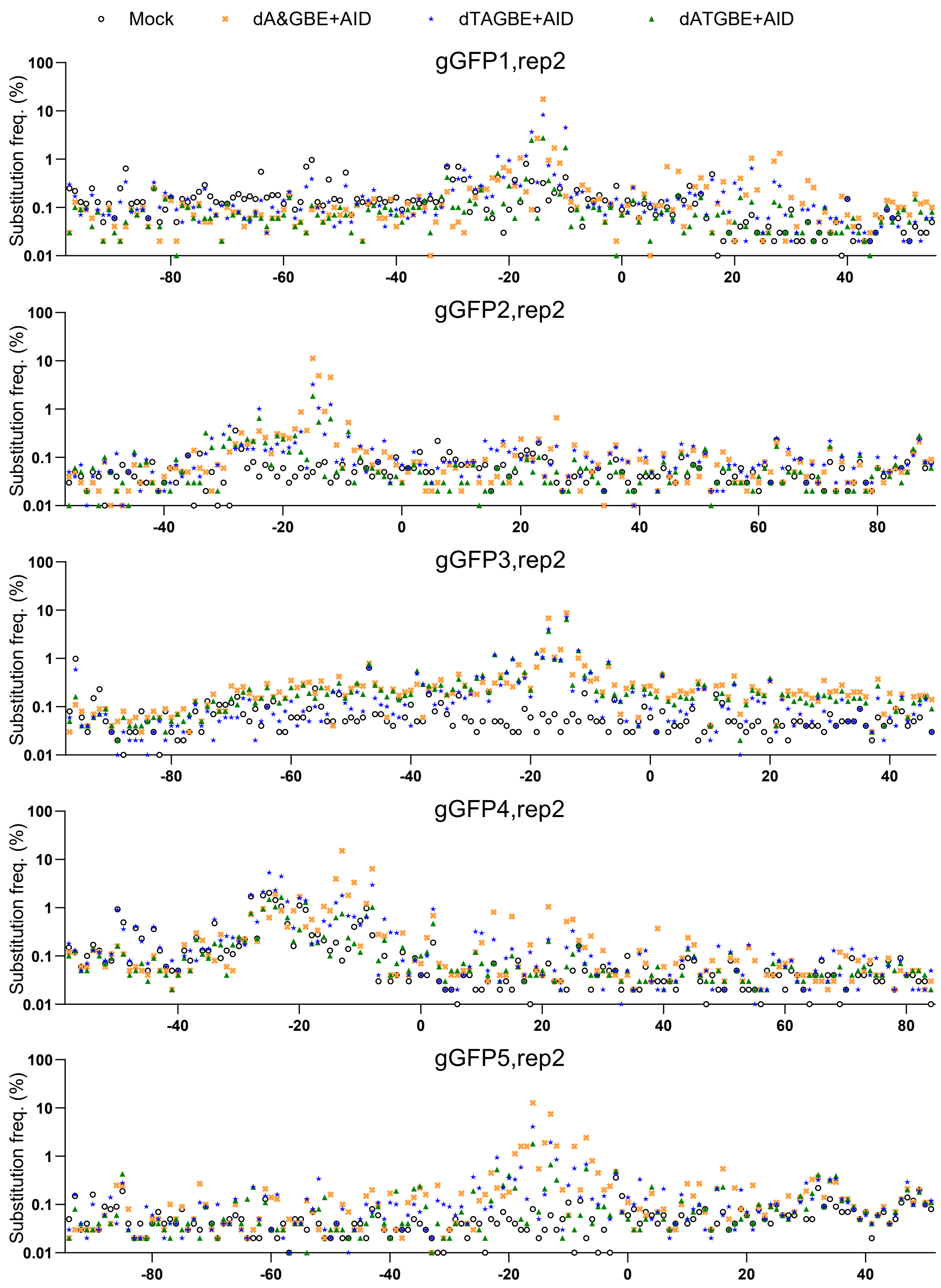

Supplementary Fig. 14 | Distribution of base substitution frequencies along the GFP sequence. Frequency (freq.) at each base was calculated as the percentage of reads containing substitutions relative to the total reads covering that base (%). Each dot represents a nucleotide. Data from three independent biological replicates are shown. Source data are provided as a Source Data file.

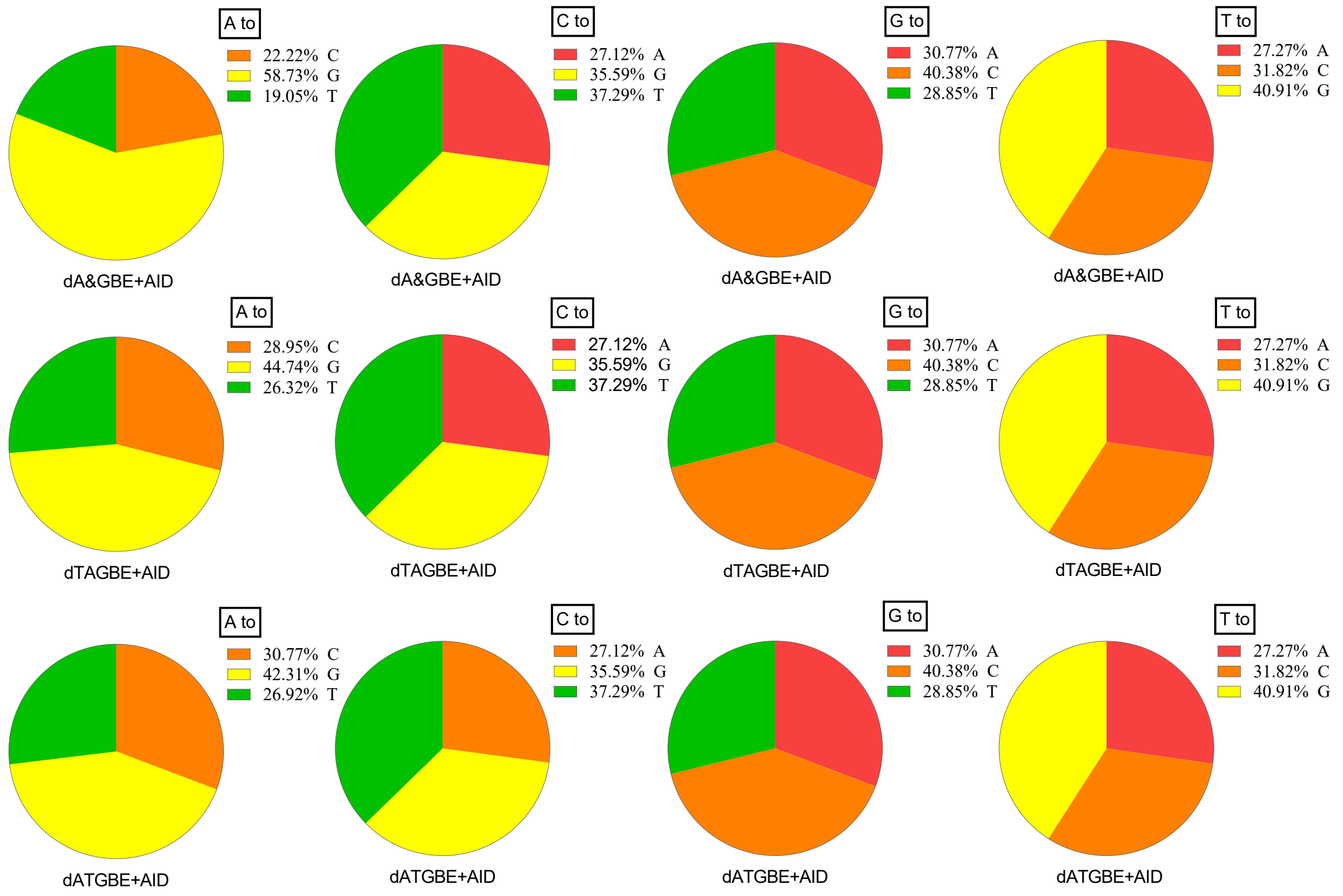

Supplementary Fig. 15 | Proportions of A, C, G, and T mutated to other bases for each group. a and b are analyzed from Fig. 5 respectively. Data are from three independent experiments. Source data are provided as a Source Data file.

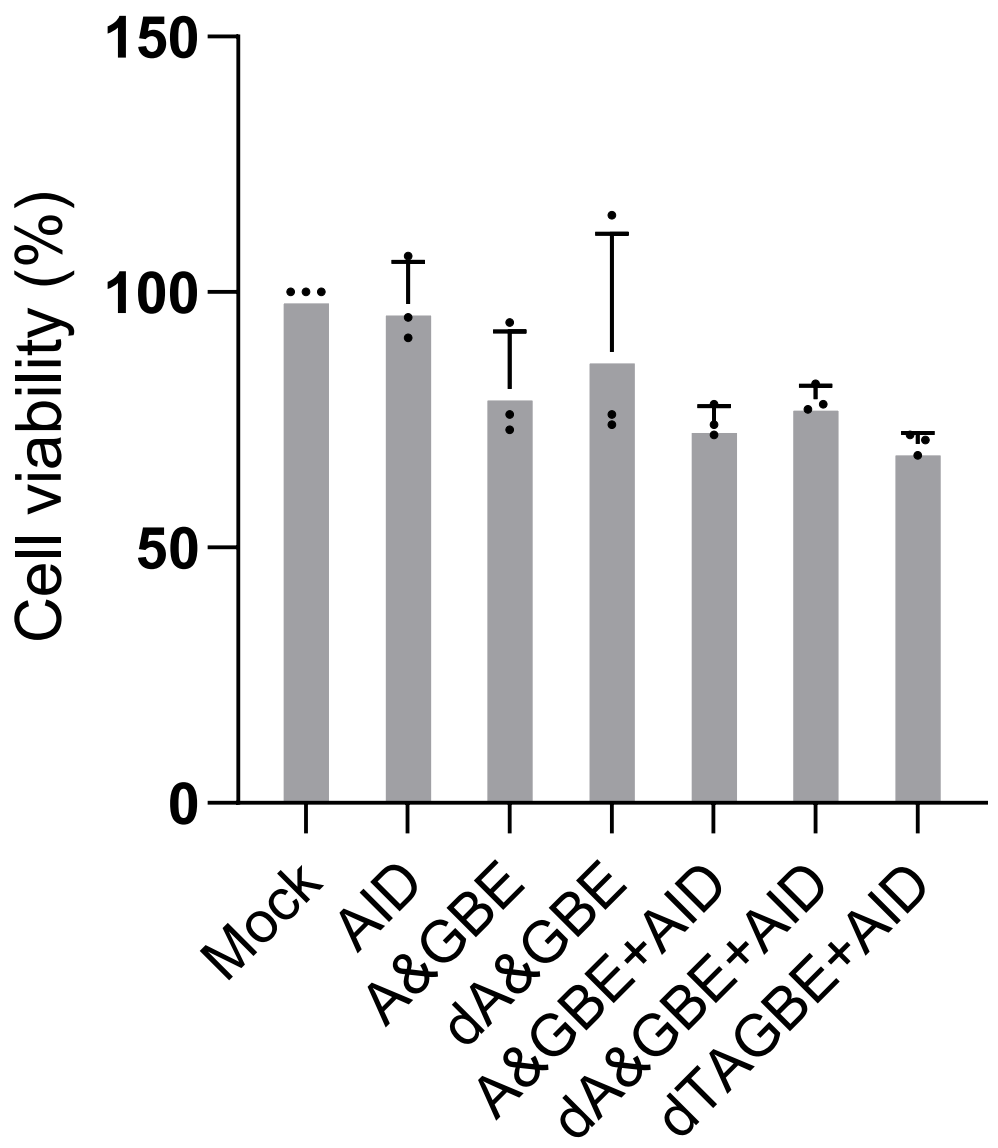

Supplementary Fig. 16 | Comparison of cell viability among base editors at 72 h. gRNA targeting GFP site 1 (gGPF1) was used. Cell viability was analyzed using CCK-8 assay. Values were normalized to the mock group (set as 100%). Values and error bars reflect the mean and s.d. of three independent experiments. Source data are provided as a Source Data file.

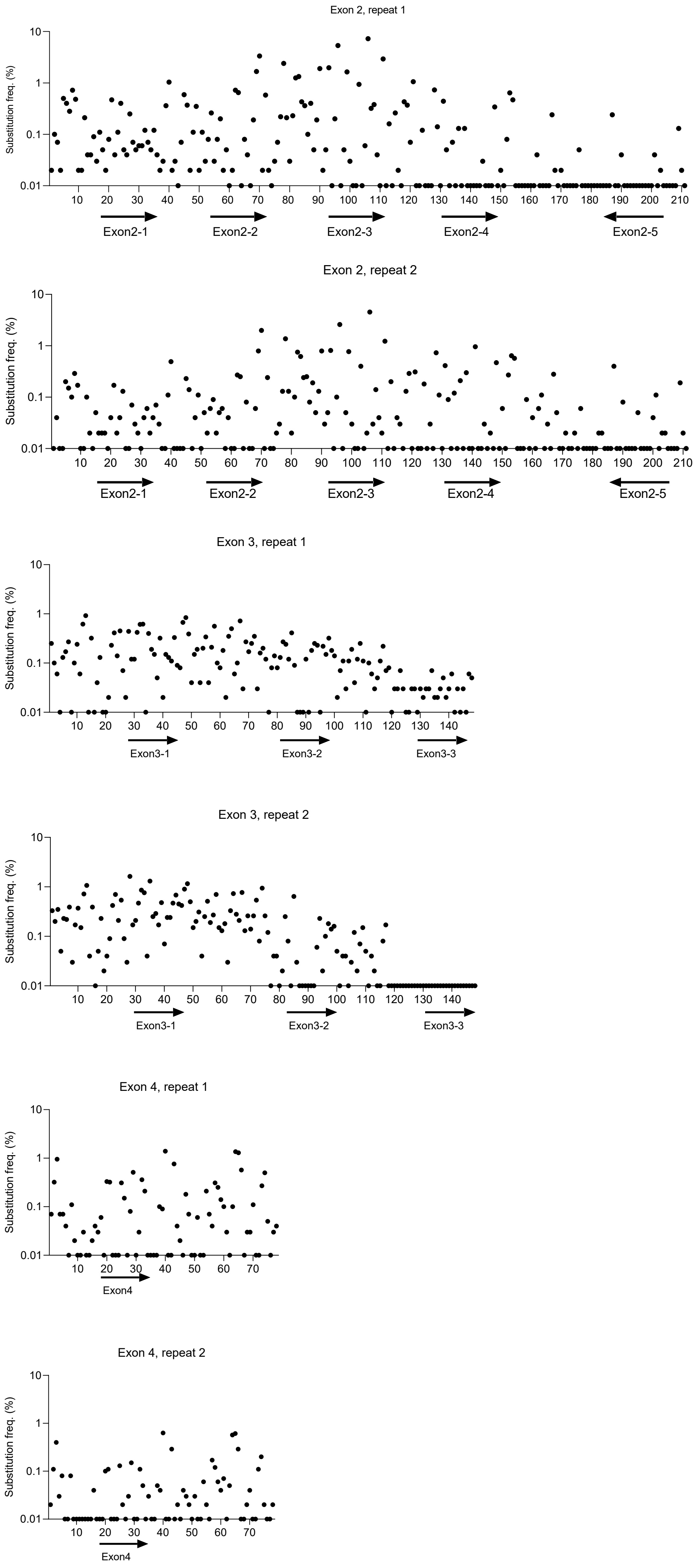
Exon 2, repeat 1
Substitution freq. (%)
10
1
0.1
0.01
10 20 30 40 50 60 70 80 90 100 110 120 130 140 150 160 170 180 190 200 210
Exon2-1
Exon2-2
Exon2-3
Exon2-4
Exon2-5
Exon 2, repeat 2
Substitution freq. (%)
10
1
0.1
0.01
10 20 30 40 50 60 70 80 90 100 110 120 130 140 150 160 170 180 190 200 210
Exon2-1
Exon2-2
Exon2-3
Exon2-4
Exon2-5
Exon 3, repeat 1
Substitution freq. (%)
10
1
0.1
0.01
10 20 30 40 50 60 70 80 90 100 110 120 130 140
Exon3-1
Exon3-2
Exon3-3
Exon 3, repeat 2
Substitution freq. (%)
10
1
0.1
0.01
10 20 30 40 50 60 70 80 90 100 110 120 130 140
Exon3-1
Exon3-2
Exon3-3
Exon 4, repeat 1
Substitution freq. (%)
10
1
0.1
0.01
10 20 30 40 50 60 70
Exon4
Exon 4, repeat 2
Substitution freq. (%)
10
1
0.1
0.01
10 20 30 40 50 60 70
Exon4

Supplementary Fig. 17 | Distribution of base substitution frequencies along MEK1 exons 2, 3, and 4. For each nucleotide position, the substitution frequency was calculated as the percentage of reads containing substitutions relative to the total reads covering that base. The plotted frequencies are shown after subtraction of the corresponding frequencies observed in untreated A375 cells. Arrows indicate the gRNA target sites, with each arrow spanning the protospacer and pointing toward the adjacent PAM sequence. Each dot represents a single nucleotide. Data from two independent biological replicates are shown. Source data are provided as a Source Data file.

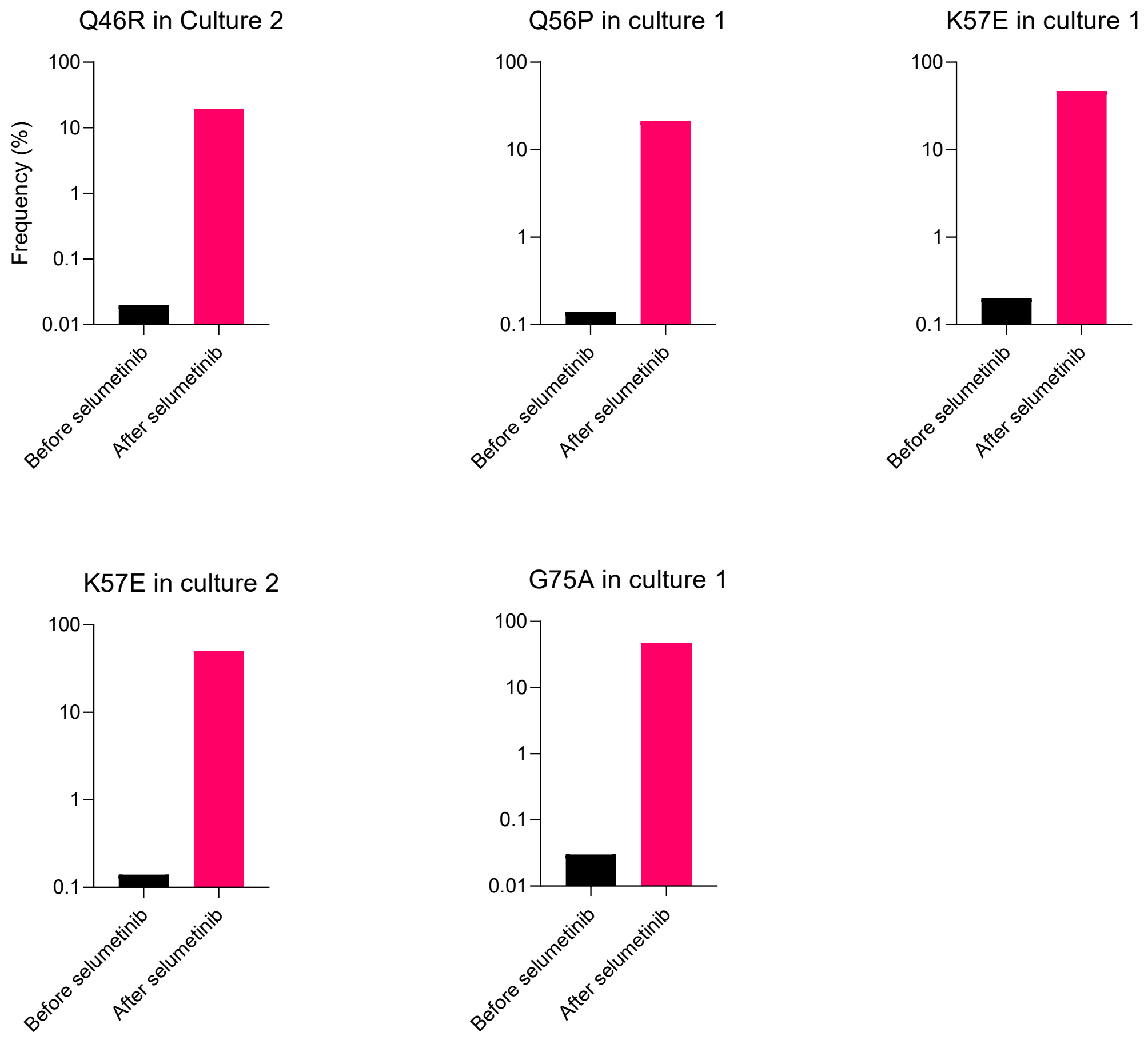

Supplementary Fig. 18 | Enrichment of MEK1 mutations following selumetinib selection. The frequency of MEK1 variants (Q46R, Q56P, K57E, and G75A) was measured in distinct cultures before and after selumetinib treatment (black and pink bars, respectively). Source data are provided as a Source Data file.
