# Supplementary material for: A dual A-to-Y and G-to-Y base editor and a four-base concurrent hypermutator in mammalian cells
Source: Nat Commun. 2026 Aug 27;17:10292. doi: 10.1038/s41467-026-77241-5 (PMC13619609; doi:10.1038/s41467-026-77241-5)
Supplement: Supplementary file 2 [file 41467_2026_77241_MOESM2_ESM.pdf]

Description of Additional Supplementary Files

File Name: Supplementary Data 1
Description: Protospacer sequences for mammalian genomic sites.

File Name: Supplementary Data 2
Description: Primers used for mammalian cell genomic DNA amplification.

File Name: Supplementary Data 3
Description: Amplicons for high-throughput sequencing analysis.

File Name: Supplementary Data 4
Description: Number of reads from amplicon sequencing
