# Supplementary material for: A dual A-to-Y and G-to-Y base editor and a four-base concurrent hypermutator in mammalian cells
Source: Nat Commun. 2026 Aug 27;17:10292. doi: 10.1038/s41467-026-77241-5 (PMC13619609; doi:10.1038/s41467-026-77241-5)
Supplement: Supplementary file 4 [file 41467_2026_77241_MOESM8_ESM.pdf]

nature portfolio

Peer Review File

A dual A-to-Y and G-to-Y base editor and a four-base concurrent hypermutator in mammalian cells

Corresponding Author: Dr Gang Wang

This file contains all reviewer reports in order by version, followed by all author rebuttals in order by version.

Version 0:

Reviewer comments:

Reviewer #1

(Remarks to the Author)
In the present manuscript, the authors integrate previously reported AYBE/GYBE routes to engineer a compact editor: a single MPG6.3-based A&GBE, further combined with MS2-MCP-recruited AID and engineered TDG to assemble triple-/quadruple-base editing modules. The study aims to broaden base diversification around target sites and is potentially useful. Nonetheless, the work reads mainly as a modular integration, and the key demonstrations for triple-/quadruple-base editing are presented primarily in a GFP-integrated HEK293T context, without systematic validation at endogenous loci or across additional cell types. Taken together, I am afraid the manuscript is not suitable for publication in its current form.

Moreover,

Safety is insufficiently characterized. The study lacks genome-wide off-target and structural-variation assessments, as well as DNA-damage and cytotoxicity readouts. Stacking multiple modules (AID/TDG/MPG) may exacerbate BER-related genotoxicity; systematic safety data are needed.

Figures 4-5 show a relatively broad editing distribution for each editor. Please report substitution frequencies in untreated/mock or empty-vector controls at the same loci and sequencing depth, and describe error-suppression steps to contextualize these patterns.

The indel bars appear to be replicated at each nucleotide position; by convention, indels in base editing studies are summarized over a defined target window rather than per nucleotide.

Sequence-context dependence. AYBE/GYBE editors show upstream/downstream sequence preferences. The manuscript does not quantify A&GBE sequence-context effects.

Reviewer #2

(Remarks to the Author)
The manuscript attempts to combine different types of deaminases and glycosylases to generate base editors with expanded mutational diversity. This concept is significant and, if successful, could provide a powerful platform to accelerate protein evolution and functional screening. However, the data presented in the current manuscript are somewhat preliminary, and the quantitative analyses are insufficient, thus failing to provide adequate support for the authors’ claims. Additional experiments and comprehensive quantitative analyses are needed, along with substantial revisions, to ensure the manuscript meets the journal’s standards and requirements.

Major comments:

1) The editing efficiencies of A-to-G/C/T and G-to-A/C/T conversions shown in Figures 1–3 do not clearly demonstrate the

advantages of A&GBE and its related variants. The authors should perform quantitative analyses of all mutation types across all tested genomic loci to provide a comprehensive overview of the editing window and activity for each mutational category.

2) The authors attempted to narrow the editing window of AYBE and A&GBE using TadA mutants and claimed that their variants exhibited improved window restriction. However, the current editing profiles at six genomic loci do not provide sufficient evidence to support this claim. Notably, E14-AYBE appears to display a similar editing window to the original construct. The authors should further evaluate the editing profiles of AYBE and A&GBE variants at additional genomic loci, similar to the analysis presented in Figure 1 with ten loci, to support their conclusions.

3) The authors attempted to reduce indel rates by substituting nCas9 with dCas9 in AYBE and A&GBE. However, they only presented editing profiles at three genomic loci in Figure 3 and did not include statistical analyses to support their claim of having "achieved a pronounced reduction in indel rates." Moreover, at two of the three loci, dA&GBE exhibited only marginal guanine editing activity, raising concerns about whether it can be appropriately described as a base editor capable of G editing.

4) The authors aimed to develop triple-base editing tools by combining A&GBE with AID. However, based on Figures 4b–c, it appears that the combination of ABE with AID alone is sufficient to induce A, C, and G editing at both protospacer and flanking regions. Moreover, the authors reported only the overall A, C, and G editing frequencies without distinguishing among A-to-G/C/T, C-to-G/A/T, and G-to-A/C/T mutations. Such detailed classification would be important for readers to assess whether these tools truly expand mutational diversity or primarily induce the canonical A-to-G, C-to-T, and G-to-C/T substitutions.
5) The authors aimed to develop quadruple base mutating module (QBMM), and describe the results in Fig. 5. As in Fig. 4, the authors reported only the overall A, C, and G editing frequencies without distinguishing among A-to-G/C/T, C-to-G/A/T, and G-to-A/C/T mutations. A detailed classification is necessary to fully demonstrate the advantages of the QBMM tool.

6) Proof-of-concept applications of these tools, particularly the QBMM tool, are essential to demonstrate their value and to support their potential in accelerating protein evolution and functional screening.

7) The tools described in this study were tested only in HEK293T cells. Additional cell lines should be examined to validate the broader applicability of these tools.

8) The authors claim in the Abstract and Discussion that their platform could be important for disease analysis. They should provide at least a discussion of some representative diseases that could be explored using these tools.

Minor comments:
1) Fig. 1 legend b, should be "at ten genomic sites".
2) Schematics of the E4, E14, and E11 TadA variants should be included to illustrate the mutation sites in comparison with TadA8e.
3) Fig. 3 legend, "Editing of AYBE and A&GBE at six genomic sites" should be "Editing of AYBE and A&GBE at three genomic sites".
4) Fig. 5a, TDG and TadA should be depicted in different colors.

Reviewer #3

(Remarks to the Author)
The manuscript presents an interesting study on the development of CRISPR-based base editing systems, particularly focusing on the concept of quadruple base mutating modules (QBMM). While the work is potentially valuable for expanding the scope of programmable base editing, several major and minor issues should be addressed before the manuscript can be considered for publication.

Introduction
The Introduction does not sufficiently highlight the significance and novelty of the quadruple base mutating modules (QBMM). The authors should clearly articulate how QBMM differs from previous base editing systems and what specific challenges it addresses in the current field of CRISPR-based base editing.

Figure 1b
There is an inconsistency in the figure legend. The text and figure both indicate that editing efficiencies were evaluated at ten endogenous loci in HEK293T cells, whereas the legend states "five genomic sites." Please correct this discrepancy.

Indel Formation in A&GBE and AYBE Systems
I noticed that both the AYBE and A&GBE editors introduce a considerable number of indels at endogenous loci, particularly at G bases. This observation warrants further discussion. The authors should comment on the potential mechanism leading to these indels and their implications for the fidelity of the editing system.

Figure 2 Presentation
The current presentation of Figure 2 makes it difficult for readers to assess whether the proposed editors truly exhibit a

narrower editing window. I recommend revising the figure format—perhaps using a more quantitative and visually intuitive representation—to better illustrate the window of activity.

Figures 4 and 5
The workflow and experimental design underlying Figures 4 and 5 are not clearly conveyed. It would be helpful to include schematic diagrams summarizing how the system operates and how GFP-integrated cell lines were used for validation. These illustrations would improve reader comprehension and overall presentation.

Results Interpretation (Figures 4b and 5b)
The conclusions drawn in the Results section regarding Figures 4b and 5b are not well supported by the data shown. The authors should either provide clearer data representation or refine their interpretation to better align with the presented results.

Experimental Validation of QBMM
Given the central claim regarding the development of the quadruple base mutating modules (QBMM), additional experimental validation is necessary. The authors are encouraged to provide more data demonstrating the editing efficiency, precision, and applicability of QBMM across multiple genomic contexts.

Overall Evaluation
In summary, this study proposes an interesting and potentially impactful approach to broaden CRISPR base editing capabilities. However, improvements in data presentation, clarification of interpretations, and additional experimental support—particularly for QBMM—are required to strengthen the manuscript.

Version 1:

Reviewer comments:

Reviewer #1

(Remarks to the Author)
The authors have adequately addressed all my previous concerns. I have no further questions or comments.

Reviewer #2

(Remarks to the Author)
While the authors have strengthened the manuscript with additional experiments and revisions, several areas still require attention, especially the data presentation style in the main figures.

1.Much of the key data—such as the comprehensive quantitative analyses for all mutation types across all tested genomic loci—has been placed in the Supplementary Figures. The authors should consider integrating some of this data into the main figures, as the current main figures lack quantitative data, and do not adequately support the manuscript's claims.
2.The authors have plotted the cumulative frequencies of A-to-other-base and G-to-other-base substitutions across the protospacer at the edited sites, as shown in Figure 2b. They should consider displaying a direct comparison in the same panel, such as between AYBE and E14-AYBE, and between A&GBE and E14-A&GBE.
3.I still believe that, the combination of ABE with AID would be sufficient to induce A, C, and G editing at both protospacer and flanking regions, according to the data shown in Figures 4b–c. The authors should provide evidence to support or reject this.
4.To improve data clarity, the authors should label the pie charts in Figure 5d and Supplementary Figures 13 and 15 with exact percentages.
5.The authors claim that the QBMM system enabled a saturation mutagenesis screen for MEK1 inhibitor-resistance in A375 cells. However, using only nine gRNAs does not inherently guarantee saturation. To support this claim, the authors should provide direct evidence of the mutation spectrum both before and after selumetinib selection to demonstrate that saturation mutagenesis was indeed achieved.

Reviewer #3

(Remarks to the Author)
Thank you for submitting the revised manuscript and the detailed point-by-point response. I have carefully evaluated the authors’ responses and the corresponding revisions. Overall, the authors have addressed the vast majority of my previous concerns, and the manuscript has been substantially improved in terms of scientific rigor, data presentation, and clarity.
In particular, the newly added validation of the QBMM system in a disease-relevant setting (A375 cell screening for MEK1 inhibitor resistance mutations; Fig. 6) significantly strengthens the study and addresses one of the major limitations of the original submission. The inclusion of additional quantitative analyses, including cumulative frequency distributions and detailed characterization of mutation spectra, further improves the quality and interpretability of the data. The revised discussion of the conceptual novelty and positioning of QBMM is also appropriate and helpful.
Despite these substantial improvements, I believe one minor point still requires clarification before the conclusions from the functional screening experiment can be fully supported.
In Fig. 6, the authors clearly demonstrate the identification of resistance-associated alleles following drug selection. However, to more rigorously evaluate the efficiency and specificity of the QBMM-based screening strategy, it would be

valuable to provide a quantitative comparison of the frequencies of the identified resistance-conferring alleles before and after selection. Specifically, reporting the pre-selection abundance of these variants and calculating their enrichment fold after selection would allow readers to better assess the extent of functional enrichment achieved and the signal-to-noise characteristics of the screening process. Such enrichment analyses are generally expected in functional selection studies and would further strengthen the interpretation of the screening results.
Therefore, I recommend that the manuscript be accepted pending minor revision to address this point.

Version 2:

Reviewer comments:

Reviewer #2

(Remarks to the Author)
The authors have addressed nearly all my previous concerns. I have no further comments.

Reviewer #3

(Remarks to the Author)
Thank you for your thorough revisions and detailed responses. You have adequately addressed all my previous concerns. I have no further questions or comments.

Open Access This Peer Review File is licensed under a Creative Commons Attribution 4.0 International License, which permits use, sharing, adaptation, distribution and reproduction in any medium or format, as long as you give appropriate credit to the original author(s) and the source, provide a link to the Creative Commons license, and indicate if changes were made.
In cases where reviewers are anonymous, credit should be given to 'Anonymous Referee' and the source.
The images or other third party material in this Peer Review File are included in the article's Creative Commons license, unless indicated otherwise in a credit line to the material. If material is not included in the article's Creative Commons license and your intended use is not permitted by statutory regulation or exceeds the permitted use, you will need to obtain permission directly from the copyright holder.
To view a copy of this license, visit https://creativecommons.org/licenses/by/4.0/

REVIEWER COMMENTS

Reviewer #1 (Remarks to the Author):

In the present manuscript, the authors integrate previously reported AYBE/GYBE routes to engineer a compact editor: a single MPG6.3-based A&GBE, further combined with MS2-MCP-recruited AID and engineered TDG to assemble triple-/quadruple-base editing modules. The study aims to broaden base diversification around target sites and is potentially useful. Nonetheless, the work reads mainly as a modular integration, and the key demonstrations for triple-/quadruple-base editing are presented primarily in a GFP-integrated HEK293T context, without systematic validation at endogenous loci or across additional cell types. Taken together, I am afraid the manuscript is not suitable for publication in its current form.
We sincerely thank the reviewer for this constructive comment. To directly address the concern regarding the lack of validation at endogenous loci, we applied the quadruple-base editing modules (QBMM) to identify MEK1 inhibitor-resistance mutations in the A375 melanoma cell line. Specifically, nine guide RNAs targeting the exons of MEK1 were designed for saturation mutagenesis. From this screen, three distinct mutations were confirmed to confer resistance to the MEK1 inhibitor selumetinib. This successful identification of functionally relevant mutations in a therapeutically important gene provides compelling evidence for the editing efficiency and practical utility of QBMM in an endogenous disease-relevant genomic context. These data have been added as Fig. 6 in the revised manuscript.

Moreover,

Safety is insufficiently characterized. The study lacks genome-wide off-target and structural-variation assessments, as well as DNA-damage and cytotoxicity readouts. Stacking multiple modules (AID/TDG/MPG) may exacerbate BER-related genotoxicity; systematic safety data are needed.
Thank you for your thoughtful and constructive comments regarding the safety characterization of our base editors. In response to your suggestion, we have now compared the cell viability among different base editors. These data are presented in Supplementary Fig. 16 and described in the Results section (“Comparison of cell viability among base editors”).

Regarding genome-wide off-target and structural variation assessments, we carefully considered the necessity of these experiments. Since no additional mutations were introduced into the base editor constructs (A&GBE, dA&GBE, dTAGBE) compared to previously characterized editors, we speculate that no significant differences would arise. Therefore, we did not perform these experiments in the current study. We acknowledge, however, that this is a limitation, and we plan

to include systematic off-target and structural variation analyses in future work when further optimizing or applying these editors.

We also recognize your important concern that stacking multiple modules (AID, TDG, MPG) might exacerbate BER-related genotoxicity. While our cell viability assay provides a preliminary cytotoxicity readout, we agree that more systematic safety data—including direct DNA damage and genotoxicity assessments—are needed. We have noted this as a key direction for our follow-up studies.

We thank you again for pushing us to improve the safety evaluation of our system.

Figures 4-5 show a relatively broad editing distribution for each editor. Please report substitution frequencies in untreated/mock or empty-vector controls at the same loci and sequencing depth, and describe error-suppression steps to contextualize these patterns.

Thank you for this important comment. As shown in Figures 4b–5b, a mock control group was already included, and substitution frequencies at the same loci were analyzed. Sequencing depth is now provided in Supplementary Data-Reads number.

To minimize sequencing errors and PCR-induced artifacts, we took the following steps: High-fidelity PCR amplification using Phanta® Max Super-Fidelity DNA Polymerase (Vazyme) with minimized cycle numbers; dual-indexed barcoding to reduce index cross-talk; stringent quality filtering of raw reads (Q≥30), adapter trimming. These measures ensure that the observed patterns are not confounded by technical artifacts.

We have also added this detailed information in the Methods section and Legend. We thank the reviewer for this valuable suggestion.

The indel bars appear to be replicated at each nucleotide position; by convention, indels in base editing studies are summarized over a defined target window rather than per nucleotide.

We thank the reviewer for this constructive suggestion. In the revised manuscript, we have updated Figs. 1–3 so that indels are now summarized over a defined target window rather than displayed at each individual nucleotide position, in accordance with the standard convention in base editing studies. Indel data are now presented separately as Supplementary Fig. 4 (for Fig. 1), Supplementary Fig. 6 (for Fig. 2), and Supplementary Fig. 9 (for Fig. 3).

Sequence-context dependence. AYBE/GYBE editors show upstream/downstream sequence preferences. The manuscript does not quantify A&GBE sequence-context effects.

We thank the reviewer for this insightful suggestion. In the revised manuscript, we have performed a quantitative analysis of the upstream and downstream sequence

preferences for AYBE, GYBE, and A&GBE. These results are now presented in Supplementary Fig. 1.

Reviewer #2 (Remarks to the Author):

The manuscript attempts to combine different types of deaminases and glycosylases to generate base editors with expanded mutational diversity. This concept is significant and, if successful, could provide a powerful platform to accelerate protein evolution and functional screening. However, the data presented in the current manuscript are somewhat preliminary, and the quantitative analyses are insufficient, thus failing to provide adequate support for the authors' claims. Additional experiments and comprehensive quantitative analyses are needed, along with substantial revisions, to ensure the manuscript meets the journal's standards and requirements.

We thank the reviewer for this suggestion. In the revised manuscript, we have performed comprehensive quantitative analyses for the data presented in Figs. 1–5. The corresponding results have been added as Supplementary Figs. 1-4 (for Fig. 1), Fig. 2b and Supplementary Figs. 5, 6 (for Fig. 2), Supplementary Figs. 7-10 (for Fig. 3), Supplementary Figs. 12, 13 (for Fig. 4), and Supplementary Figs. 14, 15 (for Fig. 5). These quantitative data provide solid support for our conclusions and address the reviewer's concern regarding the rigor of the analysis.

Major comments:

1) The editing efficiencies of A-to-G/C/T and G-to-A/C/T conversions shown in Figures 1–3 do not clearly demonstrate the advantages of A&GBE and its related variants. The authors should perform quantitative analyses of all mutation types across all tested genomic loci to provide a comprehensive overview of the editing window and activity for each mutational category.

We thank the reviewer for this insightful suggestion. In the revised manuscript, we have performed comprehensive quantitative analyses across all tested genomic loci for the data presented in Figs. 1–3. These analyses provide a detailed overview of the editing window and activity for each mutational category. The corresponding results have been added as Supplementary Figs. 2, 3 and 4b (for Fig. 1), Fig. 2b and Supplementary Fig. 6c (for Fig. 2), Supplementary Figs. 8, 9b (for Fig. 3), which more clearly illustrate the editing performance.

2) The authors attempted to narrow the editing window of AYBE and A&GBE using TadA mutants and claimed that their variants exhibited improved window restriction. However, the current editing profiles at six genomic loci do not provide sufficient evidence to support this claim. Notably, E14-AYBE appears to display a similar editing window to the original construct. The authors should

further evaluate the editing profiles of AYBE and A&GBE variants at additional genomic loci, similar to the analysis presented in Figure 1 with ten loci, to support their conclusions.

We thank the reviewer for this valuable suggestion. In response, we have evaluated the editing profiles at six additional genomic loci (see Fig. 2a and Supplementary Fig. 5). Furthermore, to enable a more direct comparison of the editing windows among AYBE, E14-AYBE, A&GBE, and E14-A&GBE, we have plotted the cumulative frequencies of A-to-other-bases and G-to-other-bases substitutions across the protospacer at the edited sites (see Fig. 2b). These additional data demonstrate that the variants exhibit narrowed editing windows compared with the original editors, supporting our conclusions.

3) The authors attempted to reduce indel rates by substituting nCas9 with dCas9 in AYBE and A&GBE. However, they only presented editing profiles at three genomic loci in Figure 3 and did not include statistical analyses to support their claim of having "achieved a pronounced reduction in indel rates." Moreover, at two of the three loci, dA&GBE exhibited only marginal guanine editing activity, raising concerns about whether it can be appropriately described as a base editor capable of G editing.

We thank the reviewer for raising this important point. In response, we have now expanded the analysis to six genomic loci (see Fig. 3 and Supplementary Fig. 7), and provided the cumulative indel frequencies across these loci to statistically support the significant reduction in indel rates (see Supplementary Fig. 9). Regarding the concern about the guanine editing activity of dA&GBE, we have additionally quantified the frequencies of G→T, G→C, and G→A editing at sites targeted by AYBE, dAYBE, GYBE, dGYBE, A&GBE, and dA&GBE (Supplementary Fig. 8). These data show that while dA&GBE exhibits attenuated G editing at some loci, its broad mutational spectrum supports its primary utility as a hypermutator rather than a conventional base editor for precise G conversion.

4) The authors aimed to develop triple-base editing tools by combining A&GBE with AID. However, based on Figures 4b–c, it appears that the combination of ABE with AID alone is sufficient to induce A, C, and G editing at both protospacer and flanking regions. Moreover, the authors reported only the overall A, C, and G editing frequencies without distinguishing among A-to-G/C/T, C-to-G/A/T, and G-to-A/C/T mutations. Such detailed classification would be important for readers to assess whether these tools truly expand mutational diversity or primarily induce the canonical A-to-G, C-to-T, and G-to-C/T substitutions.

We thank the reviewer for this constructive suggestion. In response, we have reanalyzed our data and now provide a detailed breakdown of substitution types for the triple-base editing tools shown in Fig. 4. Specifically, we generated pie charts showing the proportions of A, C, G, and T converted to each possible alternative base (see Supplementary Fig. 13). This detailed classification allows

readers to assess the full spectrum of mutations induced by our triple-base editing tools, including both canonical and non-canonical substitutions, induced by A&GBE+AID and dA&GBE+AID.

5) The authors aimed to develop quadruple base mutating module (QBMM), and describe the results in Fig. 5. As in Fig. 4, the authors reported only the overall A, C, and G editing frequencies without distinguishing among A-to-G/C/T, C-to-G/A/T, and G-to-A/C/T mutations. A detailed classification is necessary to fully demonstrate the advantages of the QBMM tool.

We thank the reviewer for this constructive suggestion. In response, we have reanalyzed our data and now provide a detailed breakdown of substitution types for the QBMM tools presented in Fig. 5. Specifically, we generated pie charts showing the proportions of A, C, G, and T converted to each possible alternative base (see Supplementary Fig. 15). This detailed classification allows readers to assess the full spectrum of mutations induced by our quadruple-base editing modules, including both canonical and non-canonical substitutions.

6) Proof-of-concept applications of these tools, particularly the QBMM tool, are essential to demonstrate their value and to support their potential in accelerating protein evolution and functional screening.

Thank you for this valuable suggestion. To demonstrate the proof-of-concept application of our tools, particularly the QBMM system, for accelerating protein evolution and functional screening, we performed a saturation mutagenesis screen to identify MEK1 inhibitor-resistance mutations in the A375 melanoma cell line. Specifically, we designed nine guide RNAs targeting the exons of MEK1 and successfully identified three distinct mutations that confer resistance to the MEK1 inhibitor selumetinib. This therapeutically relevant application demonstrates that QBMM can directly facilitate the discovery of functionally significant mutations, highlighting its potential as a powerful platform for protein engineering and high-throughput functional screening. These data have been included in the revised manuscript (Fig. 6).

7) The tools described in this study were tested only in HEK293T cells. Additional cell lines should be examined to validate the broader applicability of these tools.

We thank the reviewer for this important suggestion. To validate the broader applicability of our tools beyond HEK293T cells, we applied the QBMM system in the A375 melanoma cell line to identify MEK1 inhibitor-resistance mutations. Nine guide RNAs were designed to target the exons of MEK1, and we successfully identified three distinct mutations conferring resistance to the MEK1 inhibitor selumetinib. This successful application in an additional disease-relevant cell line demonstrates that our tools function effectively across different genomic and cellular contexts. These results have been included in the revised manuscript (Fig. 6) to further support the generalizability of our approach.

8) The authors claim in the Abstract and Discussion that their platform could be important for disease analysis. They should provide at least a discussion of some representative diseases that could be explored using these tools.
We thank the reviewer for this suggestion. In the revised Discussion, we have expanded on the disease-related applications by adding the following paragraph, which illustrates how QBMM can facilitate disease analysis through representative examples:

"QBMM therefore provides unique opportunities for disease analysis. For instance, it can recapitulate multi-nucleotide variants found in cancer (e.g., concurrent mutations in oncogenes and tumor suppressors) or in neurological disorders such as Charcot-Marie-Tooth disease. It also enables saturation mutagenesis to functionally annotate variants of uncertain significance in disease-linked genes, such as MYH7 in cardiomyopathies. These capabilities bridge unbiased hypermutation with clinically relevant disease analysis."

Minor comments:
1) Fig. 1 legend b, should be "at ten genomic sites".
We thank the reviewer for noting this error. The legend has been corrected to "at 12 genomic sites" in the revised manuscript, as two more genomics sites were added.

2) Schematics of the E4, E14, and E11 TadA variants should be included to illustrate the mutation sites in comparison with TadA8e.
We thank the reviewer for this helpful suggestion. We have now added schematics comparing the E4-, E14-, and E11-TadA variants with TadA8e, with the mutated residues clearly highlighted. The corresponding AYBE and A&GBE fusion constructs incorporating each variant are also illustrated. These diagrams are presented in Supplementary Fig. 11.

3) Fig. 3 legend, "Editing of AYBE and A&GBE at six genomic sites" should be "Editing of AYBE and A&GBE at three genomic sites".
We thank the reviewer for pointing out this discrepancy. Upon verification, we confirm that the legend should read "six genomic sites," because we have added three additional loci in the revised Fig. 3 (totaling six sites). The legend of Fig. 3 and the corresponding description in the manuscript have been corrected accordingly.

4) Fig. 5a, TDG and TadA should be depicted in different colors.
We thank the reviewer for this suggestion. Fig. 5a has been revised to depict TDG and TadA in different colors for clear distinction.

Reviewer #3 (Remarks to the Author):

The manuscript presents an interesting study on the development of CRISPR-based base editing systems, particularly focusing on the concept of quadruple base mutating modules (QBMM). While the work is potentially valuable for expanding the scope of programmable base editing, several major and minor issues should be addressed before the manuscript can be considered for publication.

We thank the reviewer for the positive assessment of our work and for recognizing the potential value of the quadruple base mutating modules (QBMM) in expanding programmable base editing. In the revised manuscript, we have carefully addressed all the major and minor concerns raised. Specifically, we have added experimental validation of QBMM in the A375 melanoma cell line (Fig. 6), performed comprehensive quantitative analyses across all genomic loci (Supplementary Figs. 1–15), improved data presentation for clarity, and refined our interpretations throughout. We believe these revisions have substantially strengthened the manuscript, and we hope it now meets the standards for publication.

Introduction

The Introduction does not sufficiently highlight the significance and novelty of the quadruple base mutating modules (QBMM). The authors should clearly articulate how QBMM differs from previous base editing systems and what specific challenges it addresses in the current field of CRISPR-based base editing.

We sincerely thank the reviewer for this insightful suggestion. We agree that the original Introduction did not adequately convey the unique significance and novelty of QBMM. In response, we have substantially revised the Introduction to clearly articulate how QBMM differs from previous base editing systems and to define the specific challenges it addresses in CRISPR-based base editing. The revised section is as follows:

"To overcome these challenges, we developed a quadruple base mutating module (QBMM), which uniquely integrates an adenine deaminase (TadA), a thymine-DNA glycosylase (TDG), a cytosine deaminase (AID), and an engineered guanine excision protein (MPG) to concurrently mutate adenine, thymine, cytosine, and guanine within a single editing window. In marked contrast to existing base editors that are confined to specific base transitions or transversions, and unlike hypermutators that exhibit strong nucleotide bias, QBMM achieves a comprehensive and well-balanced mutational spectrum across all four DNA bases. This design directly addresses the two critical bottlenecks in CRISPR-based base editing—limited editing scope and skewed mutational profiles—and fulfills the long-standing need for a mutator that simultaneously delivers high mutation rates and unbiased diversification. QBMM therefore serves as a transformative platform for directed evolution and protein engineering in mammalian cells."

Figure 1b

There is an inconsistency in the figure legend. The text and figure both indicate that editing efficiencies were evaluated at ten endogenous loci in HEK293T cells, whereas the legend states “five genomic sites.” Please correct this discrepancy.
We thank the reviewer for pointing out this inconsistency. We have corrected the legend for Fig. 1b to “12 genomic sites” to reflect the total number of loci tested in the revised manuscript (the original ten sites plus two additional sites). This resolves the discrepancy between the figure, text, and legend.

Indel Formation in A&GBE and AYBE Systems
I noticed that both the AYBE and A&GBE editors introduce a considerable number of indels at endogenous loci, particularly at G bases. This observation warrants further discussion. The authors should comment on the potential mechanism leading to these indels and their implications for the fidelity of the editing system.
We thank the reviewer for raising this important point. The mechanism of indel formation in base editors that rely on glycosylase-mediated base excision has been well-documented in prior studies on AYBE and GYBE, where the generation of abasic sites followed by AP endonuclease cleavage can combine with nCas9-induced nicks to produce DNA double-strand breaks. Consistent with this mechanism, we observed that A&GBE induces particularly high indel frequencies at guanine bases, likely reflecting the robust excision activity of MPG6.3 toward both deoxyinosine (from A deamination) and guanine. We have now added a brief description with relevant citations in the Results (line97-104).

Figure 2 Presentation
The current presentation of Figure 2 makes it difficult for readers to assess whether the proposed editors truly exhibit a narrower editing window. I recommend revising the figure format—perhaps using a more quantitative and visually intuitive representation—to better illustrate the window of activity.
We sincerely thank the reviewer for this constructive suggestion. To provide a more quantitative and visually intuitive representation of the editing window, we have revised Fig.2 to display the cumulative frequencies of A-to-other-base and G-to-other-base conversions along the protospacer at the edited sites (Fig. 2b). This format allows for a direct assessment of the editing activity distribution and facilitates comparison of the window width among different editors. We believe this revision effectively addresses the reviewer's concern.

Figures 4 and 5
The workflow and experimental design underlying Figures 4 and 5 are not clearly conveyed. It would be helpful to include schematic diagrams summarizing how the system operates and how GFP-integrated cell lines were used for validation. These illustrations would improve reader comprehension and overall presentation.
We thank the reviewer for this constructive suggestion. To provide a more quantitative and visually intuitive representation of the editing window, we have revised Fig. 2 to display the cumulative frequencies of A-to-other-base and

G-to-other-base conversions along the protospacer at the edited sites (Fig. 2b). This format enables direct assessment of the editing activity distribution and facilitates comparison of the window width among different editors.

Results Interpretation (Figures 4b and 5b)

The conclusions drawn in the Results section regarding Figures 4b and 5b are not well supported by the data shown. The authors should either provide clearer data representation or refine their interpretation to better align with the presented results.

We thank the reviewer for this valuable feedback. To better support the conclusions drawn, we have revised Figures 4b and 5b by presenting base substitution frequencies at single-base resolution along the protospacer and flanking regions. In addition, we have updated the labeling for each base editor to ensure the traces are clearly distinguishable. These refinements improve the clarity of the data representation and strengthen the alignment between the presented results and our interpretation.

Experimental Validation of QBMM

Given the central claim regarding the development of the quadruple base mutating modules (QBMM), additional experimental validation is necessary. The authors are encouraged to provide more data demonstrating the editing efficiency, precision, and applicability of QBMM across multiple genomic contexts.

We thank the reviewer for this insightful suggestion. To further validate the performance and applicability of the quadruple base mutating modules (QBMM), we applied the QBMM system to identify MEK1 inhibitor-resistance mutations in the A375 melanoma cell line. Specifically, we designed nine gRNAs targeting the exons of MEK1 and performed a saturation mutagenesis screen. From this screen, we identified three distinct mutations that confer resistance to the MEK1 inhibitor selumetinib. This successful identification of functionally relevant mutations in a therapeutically important gene provides strong experimental evidence supporting the editing efficiency, precision, and practical utility of QBMM in a disease-relevant genomic context. These results have been included in the revised manuscript (Fig. 6) to address the reviewer’ s concern.

Overall Evaluation

In summary, this study proposes an interesting and potentially impactful approach to broaden CRISPR base editing capabilities. However, improvements in data presentation, clarification of interpretations, and additional experimental support—particularly for QBMM—are required to strengthen the manuscript.

We sincerely thank the reviewer for the thoughtful and constructive assessment of our manuscript. We greatly appreciate the recognition of the potential impact of our approach in broadening CRISPR base editing capabilities. In the revised manuscript, we have carefully addressed all the concerns raised, including

improvements in data presentation, clarification of interpretations, and the addition of experimental validation of QBMM in the A375 melanoma cell line. We believe these revisions have substantially strengthened the manuscript. We hope the revised version now meets the standards of the journal and addresses the reviewer’s expectations.

REVIEWER COMMENTS

Reviewer #1 (Remarks to the Author):

The authors have adequately addressed all my previous concerns. I have no further questions or comments.

We sincerely thank the reviewer for the positive assessment and for the constructive feedback throughout the review process.

Reviewer #2 (Remarks to the Author):

While the authors have strengthened the manuscript with additional experiments and revisions, several areas still require attention, especially the data presentation style in the main figures.

1. Much of the key data—such as the comprehensive quantitative analyses for all mutation types across all tested genomic loci—has been placed in the Supplementary Figures. The authors should consider integrating some of this data into the main figures, as the current main figures lack quantitative data, and do not adequately support the manuscript's claims.

We appreciate this suggestion. To address it, we have moved the cumulative indel frequencies from Supplementary Fig. 4 and Supplementary Fig. 9 into the main figures, now shown as Fig. 1c and Fig. 3b, respectively. The remaining comprehensive datasets are too extensive to be accommodated within the main figures without compromising clarity; therefore, they are retained in the Supplementary Figures.

2. The authors have plotted the cumulative frequencies of A-to-other-base and G-to-other-base substitutions across the protospacer at the edited sites, as shown in Figure 2b. They should consider displaying a direct comparison in the same panel, such as between AYBE and E14-AYBE, and between A&GBE and E14-A&GBE.

We sincerely thank the reviewer for this constructive suggestion. As suggested, we have re-plotted Figure 2b to enable a direct side-by-side comparison. Specifically, we merged the data of AYBE and E14-AYBE into the same panels, and similarly combined A&GBE with E14-A&GBE (distinguished by different colors/grouping). This modification allows for a much clearer visual assessment of the E14 modification's effect at each protospacer position. The revised figure has been updated in the manuscript.

3. I still believe that, the combination of ABE with AID would be sufficient to induce A, C, and G editing at both protospacer and flanking regions, according to the data shown in Figures 4b–c. The authors should provide evidence to support or reject this.

Thanks for pointing out this opinion! I agree with you that the combination of ABE with AID would be sufficient to induce A, C, and G editing at both protospacer and flanking regions. However, we combined AYBE, GYBE and AID induced A to other base instead of A to G only by ABE.

We are grateful for the reviewer's thoughtful suggestion. We agree that, in theory, the combination of a conventional ABE with AID could potentially induce A, C, and G editing at both protospacer and flanking regions. However, we would like to clarify that our study adopted A&GBE (a combination of AYBE and GYBE) rather than the standard ABE. The core rationale is that AYBE and GYBE are designed to enable both A-to-other-base and G-to-other-base transitions, whereas standard ABE is strictly limited to A-to-G conversion.

To directly address the request for evidence, we note that the data in Figures 4b–c are based on the A&GBE + AID system, which clearly demonstrate simultaneous editing at A, C, and G bases across protospacer and flanking regions. This is further corroborated by the detailed base transition profiles shown in Supplementary Fig. 13 (demonstrating A→G/C/T and G→A/C/T). We hope this clarifies our experimental rationale.

4. To improve data clarity, the authors should label the pie charts in Figure 5d and Supplementary Figures 13 and 15 with exact percentages.

Thank you for this helpful suggestion. To improve data clarity, we have now explicitly added the exact percentage values as direct labels within the pie charts in Figure 5d and Supplementary Figures 13 and 15 in the revised manuscript.

5. The authors claim that the QBMM system enabled a saturation mutagenesis screen for MEK1 inhibitor-resistance in A375 cells. However, using only nine gRNAs does not inherently guarantee saturation. To support this claim, the authors should provide direct evidence of the mutation spectrum both before and after selumetinib selection to demonstrate that saturation mutagenesis was indeed achieved.

We thank the reviewer for this constructive suggestion. We acknowledge that using nine gRNAs does not inherently guarantee saturation. To provide direct evidence supporting our claim, we performed high-depth sequencing of MEK1 exons 2, 3, and 4 prior to selumetinib selection (Supplementary Figure 17). The resulting base substitution frequencies revealed a comprehensive mutational spectrum covering all three targeted exons, which demonstrates that the mutagenesis was effectively saturated at the sequence level. For the post-selection analysis, we employed a lower sequencing depth; however, the clear enrichment of the identified resistant variants (Q46R, Q56P, K57E, and G75A) further corroborates that these pre-existing clones were successfully captured during the screening (Supplementary Figure 18).

Reviewer #3 (Remarks to the Author):

Thank you for submitting the revised manuscript and the detailed point-by-point response. I have carefully evaluated the authors' responses and the corresponding revisions. Overall, the authors have addressed the vast majority of my previous concerns, and the manuscript has been substantially improved in terms of scientific rigor, data presentation, and clarity.

In particular, the newly added validation of the QBMM system in a disease-relevant setting (A375 cell screening for MEK1 inhibitor resistance mutations; Fig. 6) significantly strengthens the study and addresses one of the major limitations of the original submission. The inclusion of additional quantitative analyses, including cumulative frequency distributions and detailed characterization of mutation spectra, further improves the quality and interpretability of the data. The revised discussion of the conceptual novelty and positioning of QBMM is also appropriate and helpful.

Despite these substantial improvements, I believe one minor point still requires clarification before the conclusions from the functional screening experiment can be fully supported.

In Fig. 6, the authors clearly demonstrate the identification of resistance-associated alleles following drug selection. However, to more rigorously evaluate the efficiency and specificity of the QBMM-based screening strategy, it would be valuable to provide a quantitative comparison of the frequencies of the identified resistance-conferring alleles before and after selection. Specifically, reporting the pre-selection abundance of these variants and calculating their enrichment fold after selection would allow readers to better assess the extent of functional enrichment achieved and the signal-to-noise characteristics of the screening process. Such enrichment analyses are generally expected in functional selection studies and would further strengthen the interpretation of the screening results.

Therefore, I recommend that the manuscript be accepted pending minor revision to address this point.

We sincerely thank the reviewer for the positive assessment and for the constructive feedback throughout the review process. To address the remaining point, we performed deep sequencing of the targeted MEK1 exons prior to selumetinib selection and quantified the pre- and post-selection allele frequencies of the identified resistance variants (Supplementary Figures 17 and 18). These analyses reveal robust enrichment of all four variants, confirming the high signal-to-noise ratio and screening efficiency of the QBMM platform. These data have been incorporated into the Results section.

REVIEWERS' COMMENTS

Reviewer #2 (Remarks to the Author):
The authors have addressed nearly all my previous concerns. I have no further comments.

Response:
We thank the reviewer for their positive assessment and for confirming that all previous concerns have been satisfactorily addressed.

Reviewer #3 (Remarks to the Author):
Thank you for your thorough revisions and detailed responses. You have adequately addressed all my previous concerns. I have no further questions or comments.

Response:
We are grateful for the reviewer's careful evaluation and for acknowledging the thoroughness of our previous revisions.
